# Molecular Profiling Reveals Diversity of Stress Signal Transduction Cascades in Highly Penetrant Alzheimer's Disease Human Skin Fibroblasts

**DOI:** 10.1371/journal.pone.0004655

**Published:** 2009-02-27

**Authors:** Graziella Mendonsa, Justyna Dobrowolska, Angela Lin, Pooja Vijairania, Y.-J. I. Jong, Nancy L. Baenziger

**Affiliations:** Department of Anatomy and Neurobiology, Program in Molecular Cell Biology, Division of Biology and Biomedical Sciences, Washington University, St.Louis, Missouri, United States of America; Mental Health Research Institute of Victoria, Australia

## Abstract

The serious and growing impact of the neurodegenerative disorder Alzheimer's disease (AD) as an individual and societal burden raises a number of key questions: Can a blanket test for Alzheimer's disease be devised forecasting long-term risk for acquiring this disorder? Can a unified therapy be devised to forestall the development of AD as well as improve the lot of present sufferers? Inflammatory and oxidative stresses are associated with enhanced risk for AD. Can an AD molecular signature be identified in signaling pathways for communication within and among cells during inflammatory and oxidative stress, suggesting possible biomarkers and therapeutic avenues? We postulated a unique molecular signature of dysfunctional activity profiles in AD-relevant signaling pathways in peripheral tissues, based on a gain of function in G-protein-coupled bradykinin B2 receptor (BKB2R) inflammatory stress signaling in skin fibroblasts from AD patients that results in *tau* protein Ser hyperphosphorylation. Such a signaling profile, routed through both phosphorylation and proteolytic cascades activated by inflammatory and oxidative stresses in highly penetrant familial monogenic forms of AD, could be informative for pathogenesis of the complex multigenic sporadic form of AD. Comparing stimulus-specific cascades of signal transduction revealed a striking diversity of molecular signaling profiles in AD human skin fibroblasts that express endogenous levels of mutant presenilins PS-1 or PS-2 or the Trisomy 21 proteome. AD fibroblasts bearing the PS-1 M146L mutation associated with highly aggressive AD displayed persistent BKB2R signaling plus decreased ERK activation by BK, correctible by gamma-secretase inhibitor Compound E. Lack of these effects in the homologous PS-2 mutant cells indicates specificity of presenilin gamma-secretase catalytic components in BK signaling biology directed toward MAPK activation. Oxidative stress revealed a JNK-dependent survival pathway in normal fibroblasts lost in PS-1 M146L fibroblasts. Complex molecular profiles of signaling dysfunction in the most putatively straightforward human cellular models of AD suggest that risk ascertainment and therapeutic interventions in AD as a whole will likely demand complex solutions.

## Introduction

Alzheimer's disease is a neurodegenerative disorder that affects the brain's cognitive functioning and memory retention properties [1]. Nearly 90% of all AD cases are a complex multigenic disorder (Sporadic AD) [2], while less than 5% represent familial cases (FAD) caused by highly penetrant genetic mutations in APP, PS-1 or PS-2 [3], [4]. AD brain autopsies reveal hallmarks of Aβ-bearing senile plaques, *tau*-associated neurofibrillary tangles and enhanced neuronal loss that reflect the complex series of responses comprising the AD disease process [5]. Accumulation of Aβ_1–42_ drives amyloid based AD pathology *in vivo*, and AD-related hyperphosphorylation of *tau* negatively affects the ability of *tau* to maintain microtubules, resulting in neurofibrillary tangles [1], [5], [6]. Oxidative stress and head trauma injury have been established as risk factors and early events in the development of AD [7], [8], [9].

Aberrant function of signal transduction pathways at the cellular and molecular level is associated with AD [10]. We have previously defined exaggerated signal transduction in AD patients' skin fibroblasts in response to the inflammatory neuropeptide bradykinin (BK). Levels of this nonapeptide mediator generated via a proteolytic cascade increase under environmental insults such as stroke, head trauma injury, and pain [8], [11], to initiate signaling pathways via G-protein-coupled receptors [12]. In a PKC-dependent process the BK B2 subtype receptor (BKB2R) is modulated to forms that reflect activity of a Tyr phosphorylation pathway [13]. The modulated B2 receptors respond to BK at pathophysiologic levels of 25–250 nM characteristic of tissue injury, in skin fibroblasts from persons not only having familial AD presently but those at risk for future AD due to Trisomy 21 [8]. Stimulation of *tau* phosphorylation on Ser residues is the downstream consequence of BK-induced PKC signaling in fibroblasts from AD and Trisomy 21 patients [8]. Neither the PKC-dependent BK B2R modulation nor the consequent *tau* Ser phosphorylation occurs in any normal skin fibroblasts from persons aged 17–82 [8].

In the brain, AD patients display neuronal loss affecting memory and reasoning centers in the hippocampus and cerebral cortex, respectively [1]. These regions correspondingly are the ones that most prominently display BK biology and its signaling pathways [14]. An Aβ rich environment can prompt increases in BK generation and hence activity of the BKB2R [14], making the role of inflammatory stress signaling a key focus in AD pathogenesis.

The role of oxidative stress in AD pathogenesis may center on cell cycle re-entry, apoptosis, APP processing, Aβ secretion and *tau* phosphorylation [15] as well as in the disruption of Ca^2+^ homeostasis [16]. The brain exhibits particularly high sensitivity to oxidative stress [7], [9] and AD brains reveal loss of synapses and oxidative stress damage by reactive oxygen species (ROS) [17]. In APP transgenic mice, Aβ co-localizes with several oxidative stress markers suggesting an *in vivo* link between Aβ deposition and oxidative damage [9], [18]. Oxidative stress promotes Aβ toxicity through the production of free radicals [9], [19], and the application of Aβ to neuronal cultures elevates intracellular H_2_O_2_ levels resulting in neuronal apoptotic morphology [9], [20]. Intriguingly, ROS generation is not just an outcome of the AD disease process itself but serves as a signaling mechanism and actually precedes Aβ deposition as one of the earliest events in AD pathology [16], [17], [21], [22].

**Figure 1:**
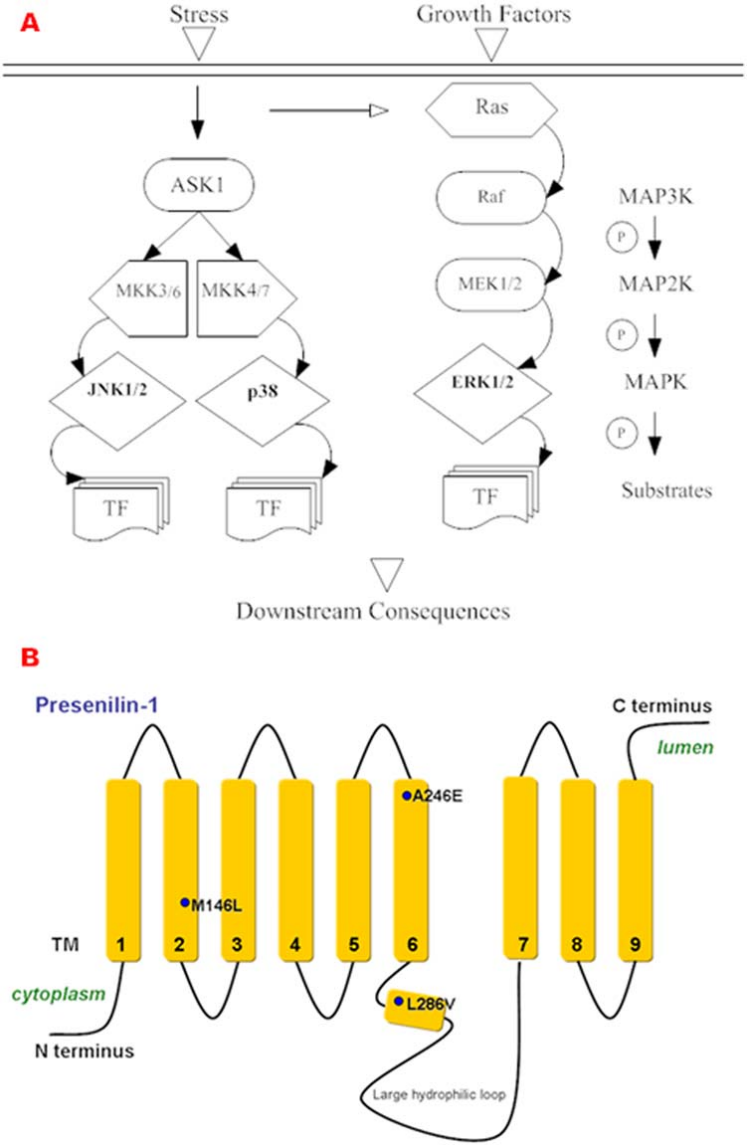
Mediators and modulators of fibroblast signaling in Alzheimer's Disease. A. Schematic of the MAPK pathway. The MAPK pathway functions by sequential phosphorylation events: MAP3K→MAP2K→MAPK→substrates. The three MAPK modules consisting of JNK1/2, p38 and ERK1/2 are both indicators and mediators in stress responses. MAPKs regulate nuclear target genes through phosphorylation of multiple transcription factor substrates (TF) as well as membrane and cytoskeletal protein targets. B. Schematic of PS-1 and sites of familial AD mutations. Presenilin-1 (PS-1) functions as a key component in the gamma-secretase complex along with participants PS-2, nicastrin (NCT), anterior pharynx-defective phenotype 1 (APH-1) and PS enhancer-2 (PEN-2) [38]. PS-1 has multiple transmembrane (TM) domains and the 3 sites of Familial AD (FAD) mutations investigated are positioned within their respective domains.

Both BK and ROS signaling activate the MAPK cascade superfamily as a downstream response pathway [23], [24], [25], [26] whose sequential phosphorylation events: MAP3K→MAP2K→MAPK [27] occur in three parallel modules (see schematic, Figure 1A) [1], [28]. Upon dual Tyr-Thr phosphorylation JNK and p38 are activated under environmental stresses such as UV radiation, heat shock, and oxidative stress, while ERK is activated by mitogens and growth factors to promote cell survival, differentiation and cell cycle regulation [9], [24]. MAPKs have been postulated to act aberrantly in the context of AD pathology [29]. JNK, p38 and ERK have been shown to phosphorylate *tau in vitro* at AD specific sites and to co-localize with *tau in vivo* [30], [31], [32].

To understand how inflammatory stress and oxidative stress signals may serve as both guideposts and vehicles for development of AD pathology, we have profiled how these stresses trigger key signal transduction cascades of the MAPK superfamily in human skin fibroblasts from established familial AD and Trisomy 21 patients and a panel of normal control fibroblasts spanning the age range of the AD and Trisomy 21 patients. (See Table 1). The AD fibroblasts of different genetic origins expressing endogenous levels of presenilins mutated at various sites (see schematic, Figure 1B adapted from [33], [34], [35], [36], [37], [38]) respond differently to stress induced by BK or H_2_O_2_ compared with one another and with normal fibroblasts. This results in unique profiles of stress-induced MAPK activation and caspase-3 cleavage, as well as downstream events such as cell death.

**Table 1:** AD and Normal Control Cell Lines.

HSF Cell Line	Genotype	Donor Age/Sex	AD Present	Onset Age of AD
7872	PS-1 M146L	53 / M	Yes	35–45
8170	PS-1 A246E	56 / M	Yes	53
8597	PS-1 L286V	49 / M	Yes	48
9908	PS-2 N141I	81 / F	Yes	40–80
11368	No PS mutations	77 / M	Yes	70
8941	Trisomy 21	19 / F	No	30–40
8942	Trisomy 21	20 / M	No	30–40
6234B	Normal	17 / M	No	n.a.#
8539	Normal	53 / M	No	n.a.
8269	Normal	82 / F	No	n.a.

HSF = Human Skin Fibroblast from Coriell Institute.

# n.a. = not applicable (Table modified from Jong, Y, et al., 2003).

## Results

### PS-1 M146L AD fibroblasts display prolonged BKB2R modulation

**Figure 2:**
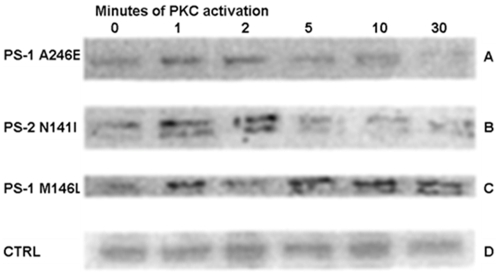
PS-1 (M146L) AD fibroblast line displays persistent BK-B2R modulation upon PKC activation. PS-1, PS-2 and control non-AD fibroblasts (CTRL) were treated with 25 nM PMA for the indicated times. B2 receptors in non-ionic detergent solubilizates were detected by immunoblotting and chemiluminescence detection. Results are representative of 3 experiments for PS-1 and control and 4 for PS-2.

We previously found that activation of PKC by 25 nM phorbol myristate acetate (PMA) or by treatment with 250 nM BK prompted modulation of the BKB2R in AD skin fibroblasts (Table 1), detected by monoclonal anti-BKB2R antibodies recognizing the modulated receptors that reflect enhanced activity of a Tyr phosphorylation pathway in the AD cells [8], [12]. Only trace, non-PKC-dependent levels of the phosphorylated receptor occur in skin fibroblasts of normal controls. We subsequently have defined how persistent this modulated receptor signal is across AD skin fibroblast lines bearing mutations in different locations along the presenilin (PS) polypeptide chain. As shown in Figure 2, the phosphorylated receptor increased rapidly in response to PKC activation, reaching a maximum of 4-fold over the basal level seen in un-stimulated AD fibroblasts at 1–2 min of phorbol ester treatment. Beyond that point, in PS-1 A246E skin fibroblasts the modulated forms of the BKB2R declined in the subsequent 5–30 min time frame (Figure 2A), and PS-1 L286V fibroblasts showed an identical decline (data not shown). A similarly transient state of modulation was evident in PS-2 N141I AD skin fibroblasts (Figure 2B). Trace levels of phosphorylated BKB2R in age-matched control fibroblasts required extensive chemiluminescent exposure to detect and did not change significantly with time of PMA treatment (Figure 2D). However, modulated receptors were notably more persistent in PS-1 M146L AD fibroblasts, exhibiting a lifetime out to 30 min of PKC activation with PMA (Figure 2C). This sustained presence of the phosphorylated receptor BKB2R receptor forms suggested a particularly enhanced aberrant signaling profile associated with this particular PS-1 mutation as expressed at endogenous levels in AD skin fibroblasts.

### Bradykinin stimulation of PS-1 (M146L) AD fibroblasts results in decreased MAPK activation relative to control fibroblasts

**Figure 3:**
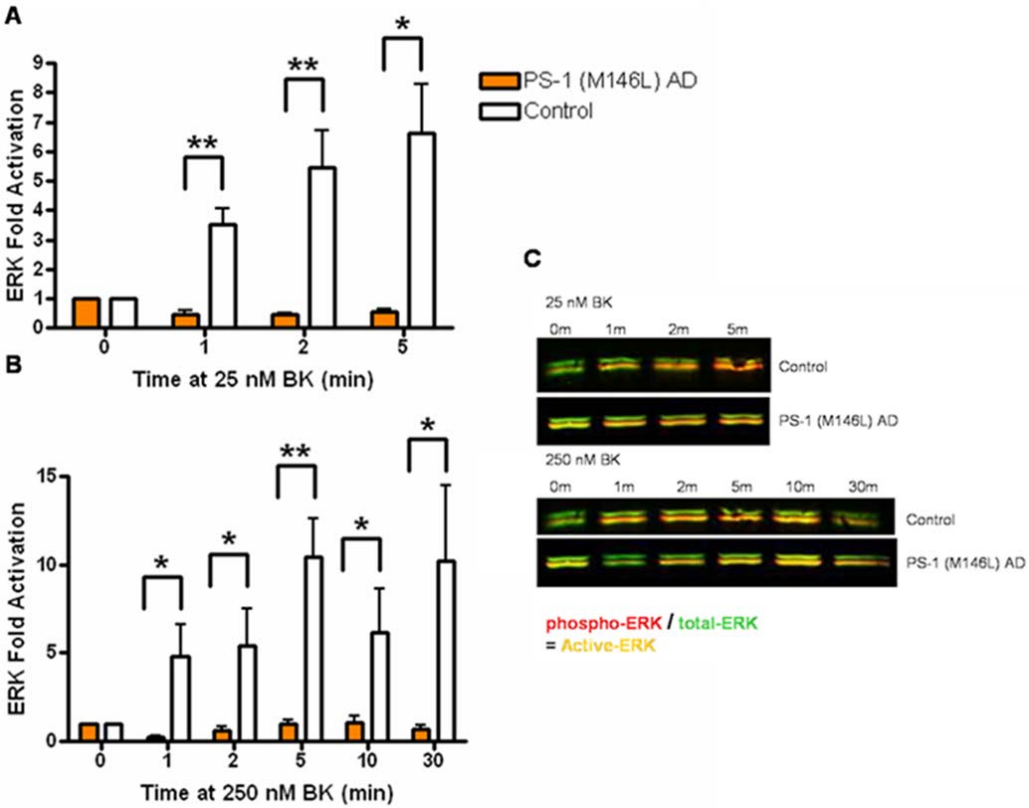
Bradykinin stimulation of PS-1 (M146L) AD fibroblasts results in decreased ERK activation relative to control fibroblasts. PS-1 (M146L) AD and control human skin fibroblasts were treated with 25 nM BK or 250 nM BK for 0–30 min, immunoblotted for active and total ERK with phospho-epitope specific and total ERKantibodies, and analyzed by both ECL and Odyssey detection. Fold activation was quantified as a percentage of basal activation in buffer treated cells (0 min) normalized to tubulin and is graphically represented for Active ERK (A–B). Representative Odyssey immunoblots show phospho-ERK in red and total-ERK in green that together in yellow display differential ERK activation profiles between PS-1 (M146L) AD and control fibroblasts induced by 25 or 250 nM BK (C). All graphs show mean±S.E. Statistical analysis was performed via t-test with *p-value<0.05 and **p<0.005; n = 4.

**Figure 4:**
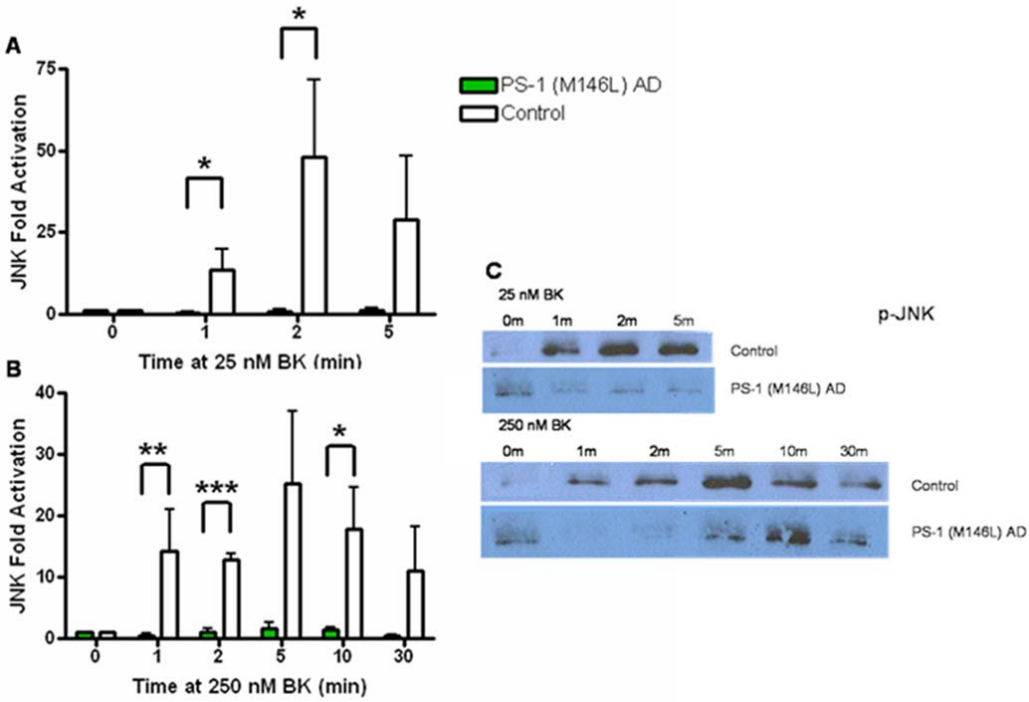
Bradykinin stimulation of PS-1 (M146L) AD fibroblasts results in decreased JNK activation relative to control fibroblasts. PS-1 (M146L) AD and control human skin fibroblasts treated with 25 nM BK (A) or 250 nM BK (B) for 0–30 min were immunoblotted for active JNK with phospho-epitope specific antibodies and analyzed by ECL. Fold activation was quantified as a percentage of basal activation in buffer treated cells (0 min) normalized to tubulin and is graphically represented for active JNK (A–B). Representative immunoblots shown display differential JNK activation profiles between PS-1 (M146L) AD and control fibroblasts (C). All graphs show mean±S.E. Statistical analysis was performed via t-test with *p-value<0.05 and ***p<0.0005; n = 4.

**Figure 5:**
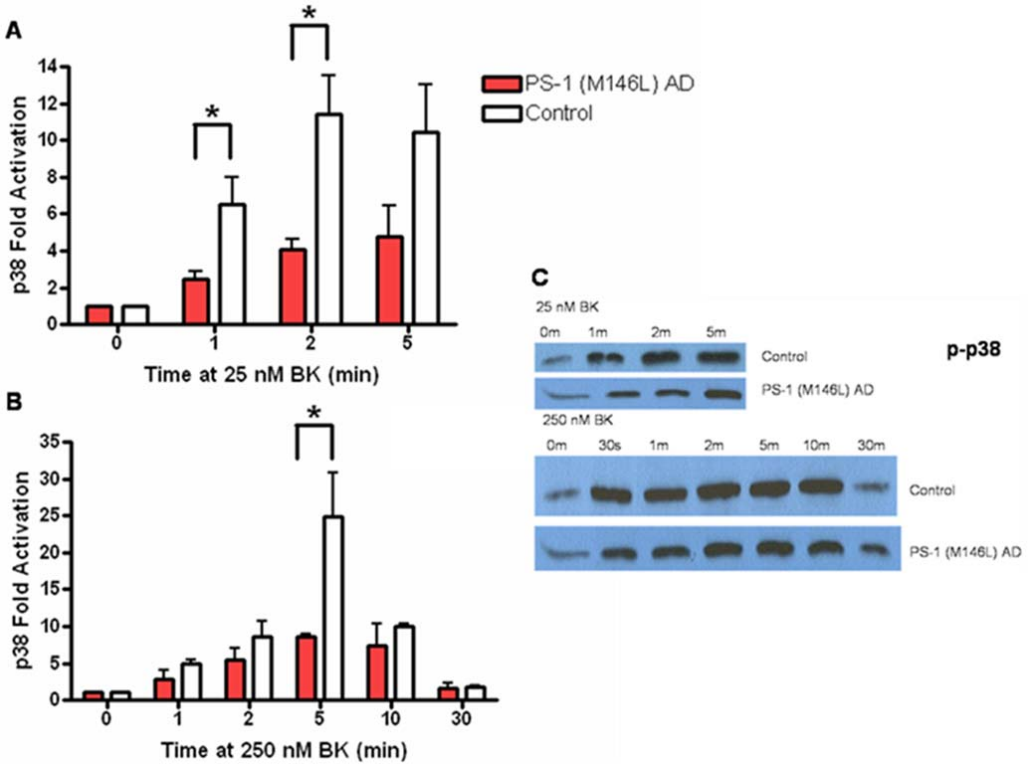
Bradykinin stimulation of PS-1 (M146L) AD fibroblasts results in decreased p38 activation relative to control fibroblasts. PS-1 (M146L) AD and control human skin fibroblasts treated with 25 nM BK (A) or 250 nM BK (B) for 0–30 min were immunoblotted for active p38 with phospho-epitope specific antibodies and analyzed by ECL. Fold activation was quantified as a percentage of basal activation in buffer treated cells (0 min) normalized to tubulin and is graphically represented for active p38 (A–B). Representative immunoblots shown display differential p38 activation profiles between PS-1 (M146L) AD and control fibroblasts (C). All graphs show mean±S.E. Statistical analysis was performed via t-test with *p-value<0.05; n = 4.

Given the increased length and hence strength of aberrant BKB2R signal transduction in the PS-1 M146L AD fibroblasts, this cell line was chosen for further study of putative downstream signaling steps beyond the BKB2R itself, focusing on the MAP kinase pathway. Normal control and PS-1 (M146L) AD human skin fibroblasts were stimulated for 0–30 min with BK concentrations of 25 or 250 nM, which respectively target the I (intermediate) and L (low) affinity phosphorylated BKB2R forms we previously characterized in AD fibroblasts [12], [13], [39], [40]. Cell lysates collected in 1% SDS were immunoblotted after SDS-PAGE using phospho-epitope specific antibodies to quantitate activity of MAPK modules (p38, ERK and JNK). All three MAPK modules were activated by both concentrations of BK. ERK, traditionally viewed as responsive to activation of growth factor receptors, was here activated by the GPCR BKB2 receptor. However, ERK displayed a lag in the onset and a reduced magnitude and duration of activation in PS-1 M146L AD fibroblasts stimulated with BK, compared to the activation response of normal control fibroblasts. Activation of ERK was substantially diminished at both 25 nM and 250 nM BK in PS-1 (M146L) AD fibroblasts (Figure 3A–C). Similarly to ERK, the stress-activated MAPK modules of JNK (Figure 4A–C) and p38 (Figure 5A–C) were both activated by both BK concentrations and showed a diminished response in the PS-1 (M146L) fibroblasts. Compared to ERK and JNK, p38 displayed a more robust activation in the AD fibroblasts, peaking at 2–5 min with 25 nM and 5–10 min with 250 nM BK stimulation and subsiding by 30 min (Figure 5A–C). Yet the magnitude of p38 activation was still significantly decreased in PS-1 (M146L) AD fibroblasts compared to normal controls. Based on the MAPK activity profiles in PS-1 (M146L) AD fibroblasts, we extended our study to BK dependent MAPK behavior in additional AD skin fibroblasts representing a variety of genetic backgrounds.

### Decreased BK responsiveness of ERK activity is specific to PS-1 FAD mutations in AD fibroblasts

**Figure 6:**
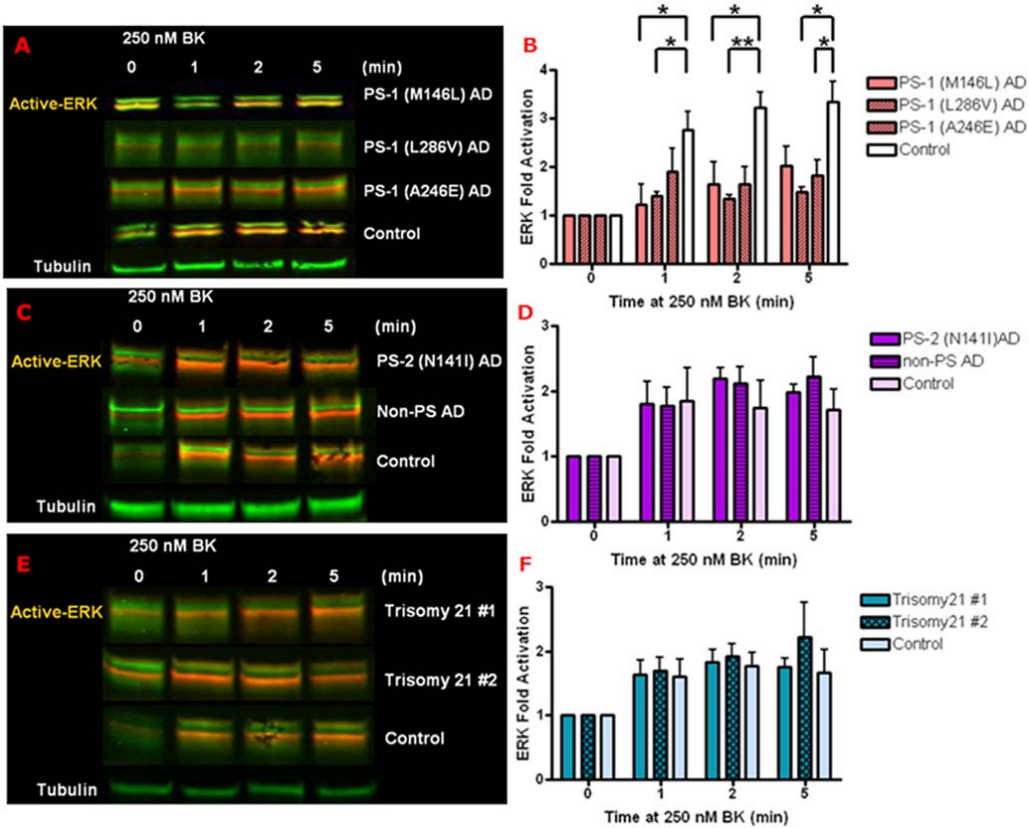
Decreased BK responsiveness of ERK activation is found only in PS-1 FAD mutant AD fibroblasts. Control human skin fibroblasts and those from AD and Trisomy 21 patients were treated with 250 nM BK for 0–5 min. Cell lysates were immunoblotted as in Figure 3 and analyzed by Odyssey, normalized to tubulin as loading control (A, C and E). BK-induced ERK activation was quantified as a percentage of basal activation in buffer treated cells (0 min). ERK activity phenotypes are graphically represented in fibroblasts with PS-1 familial AD mutation: M146L, L286V, or A246E (B), PS-2 N141I and non-PS AD fibroblasts (D) as well as AD pre-disposed Trisomy 21 fibroblasts (F). MAPK activity profiles in AD and Trisomy 21 fibroblasts were compared to age and gender matched normal controls. All graphs show mean±S.E. Statistical analysis was performed via t-test with *p-value<0.05, **p<0.005; n = 4.

BK stimulation of MAPK cascades was tested in a broad series of AD fibroblasts (Coriell Institute) derived from familial Alzheimer's disease (FAD) patients with known mutations in PS-1 or PS-2, as well as from an ApoE4 homozygote expressing no known PS mutation (non-PS) (Table 1). Also tested were fibroblasts from late adolescent Trisomy 21 Down syndrome patients at very high risk for developing AD by the age of 35 [8]. The control, AD and Trisomy 21 fibroblasts were similar in growth rate properties [8]. The MAPK activation responses of all these fibroblasts to 250 nM BK were compared with age and gender matched normal controls. All the PS-1 Familial AD human skin fibroblast lines displayed significantly decreased ERK activation during the first 5 min of BK treatment as compared to controls (Figure 6A–B). However the PS-2 and the non-PS AD fibroblasts yielded BK-stimulated ERK activation levels similar to that of the corresponding controls (Figure 6C–D). Down syndrome fibroblasts from teen-aged subjects well before the age of onset of symptomatic AD in this population also displayed BK-mediated ERK activation comparable to that of age-matched normal control fibroblasts (Figure 6E–F). Thus the decreased responsiveness of ERK activation by 250 nM BK is specifically associated with PS-1 mutant AD.

**Figure 7:**
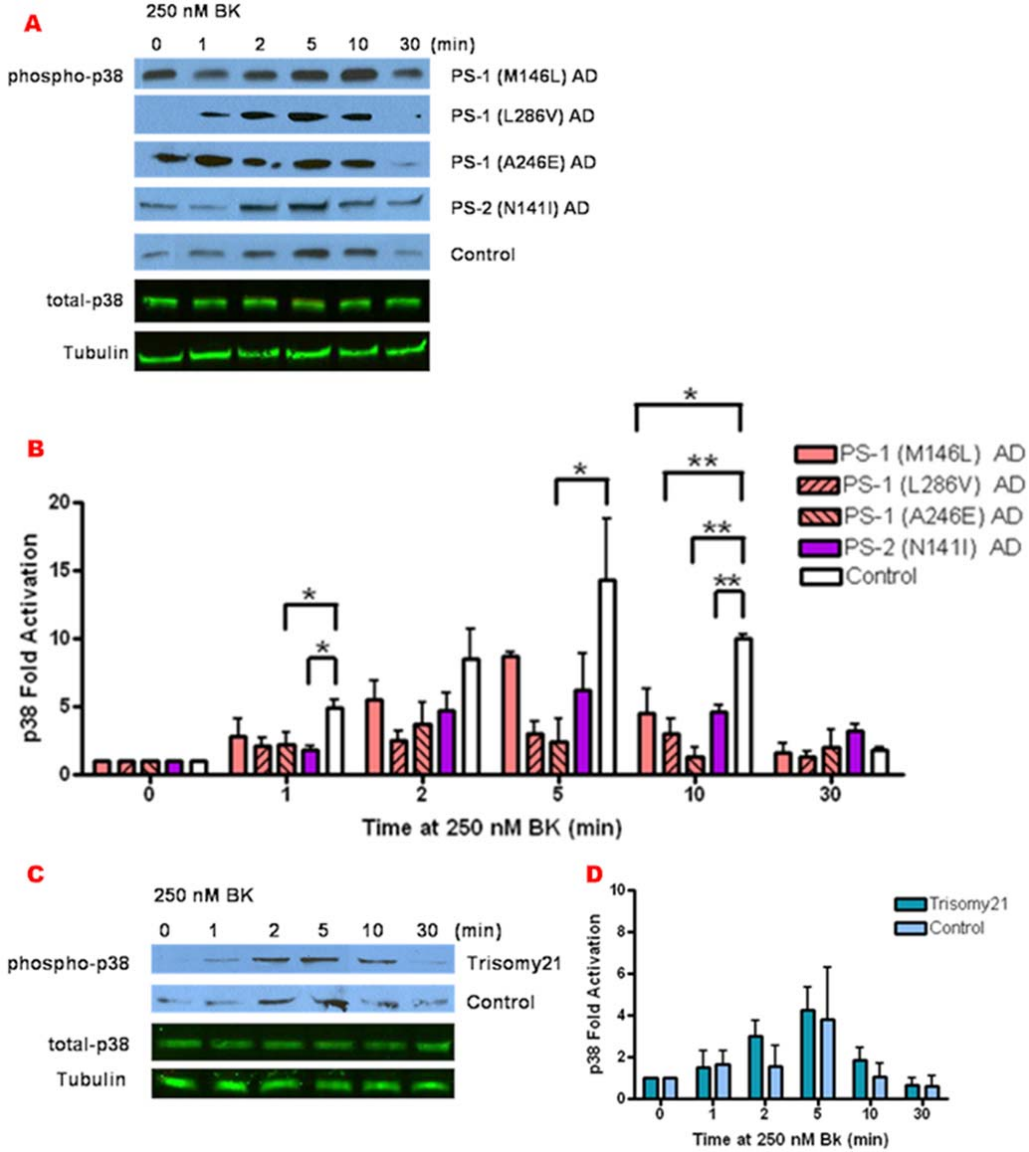
Decreased BK responsiveness of p38 activation is evident in both PS-1 and PS-2 FAD mutant AD fibroblasts. Control human skin fibroblasts and those from AD and Trisomy 21 patients were treated with 250 nM BK for 0–30 min. Cell lysates were immunoblotted for active p38 with phospho-epitope specific antibodies and analyzed by ECL, and for total p38 and tubulin loading control analyzed by Odyssey. Representative blots are shown in (A) and (C). Fold activation of p38 was quantified as a percentage of basal activation in buffer treated cells (0 min) normalized to tubulin. p38 activity phenotypes are graphically represented in fibroblasts with PS-1 familial AD mutation: M146L, L286V, or A246E and PS-2 N141I (B) as well as AD pre-disposed Trisomy 21 fibroblasts (D). MAPK activity profiles in AD and Trisomy 21 fibroblasts were compared to age and gender matched normal controls. All graphs show mean±S.E. Statistical analysis was performed via t-test with *p-value<0.05, **p<0.005; n = 3.

Although JNK activation was so low in the AD cells as to preclude in-depth analysis, we further defined the BK responsiveness of p38 activation in all of the presenilin mutant AD fibroblasts compared with normal control cells (Figure 7A–B). The presenilin mutant AD fibroblasts exhibited a variable degree of constitutive p38 activity not dependent on BK (Figure 7A, “0” lanes). However p38 activation by BK was blunted in all of the presenilin mutants relative to control fibroblasts, particularly evident at 10 min of BK treatment (Figure 7B). In contrast, Trisomy 21 fibroblast p38 activation in response to BK was comparable to that of normal controls (Figure 7C–D).

### Gamma-secretase inhibitor Compound E restores normal BK-dependent ERK activation in PS-1 (M146L) AD fibroblasts

**Figure 8:**
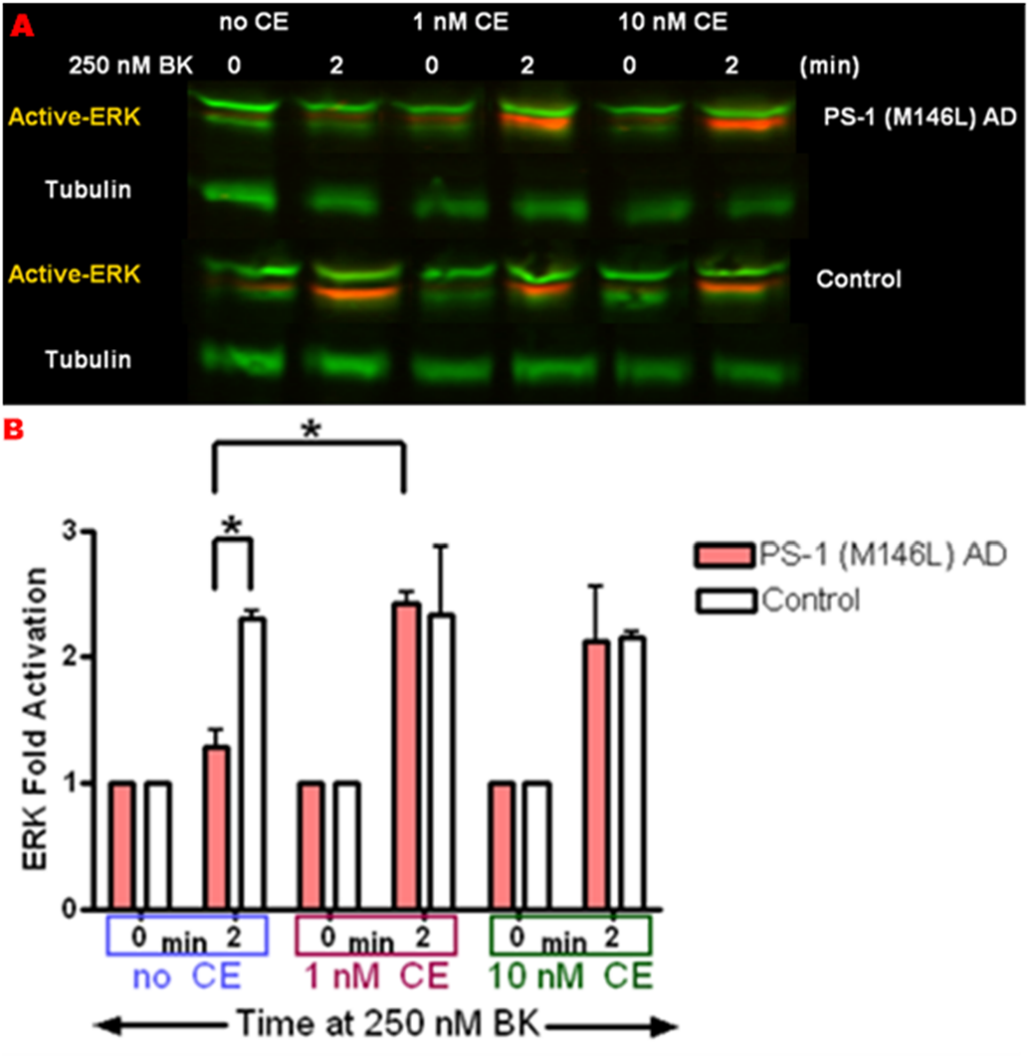
Gamma-secretase inhibitor Compound E restores normal BK-dependent ERK activation in PS-1 (M146L) AD fibroblasts. PS-1 (M146L) AD and normal control human skin fibroblasts were treated with Gamma secretase inhibitor Compound E (1 or 10 nM) or DMSO vehicle control for 24 hr and then stimulated with 250 nM BK for 0–30 min. Cell lysates were immunoblotted for ERK as in Figure 6 and analyzed by Odyssey for ERK activation normalized to Tubulin as loading control. A representative blot is shown in (A). ERK activation was quantified as a percentage of basal activation in buffer-treated cells (0 min) pre-treated with DMSO or Compound E. ERK activity phenotypes are graphically represented in control fibroblasts compared with PS-1 (M146L) AD fibroblasts (B). All graphs show mean±S.E. Statistical analysis was performed via t-test with *p-value<0.05; n = 3.

**Figure 9:**
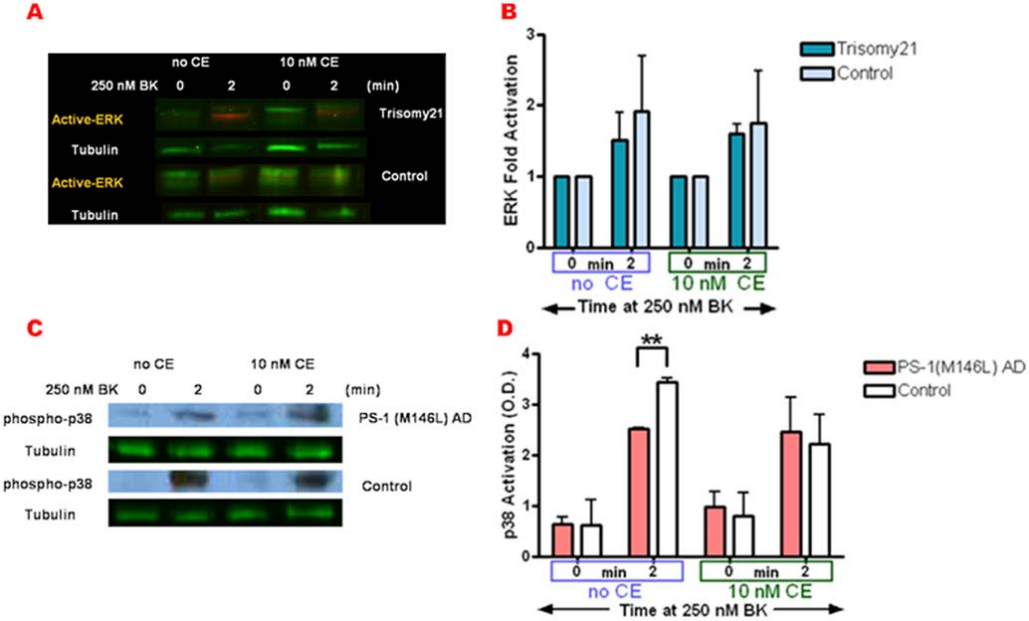
Gamma-secretase inhibitor Compound E affects BK-dependent p38 activation in PS-1 (M146L) AD fibroblasts but not ERK activation in Trisomy 21 fibroblasts. Trisomy 21 and PS-1 (M146L) AD fibroblasts plus normal control human skin fibroblasts were treated with Gamma secretase inhibitor Compound E (10 nM) or DMSO vehicle control for 24 hr and then stimulated with 250 nM BK for 0–30 min. Cell lysates were immunoblotted and analyzed as in Figures 6 and 7, by Odyssey for ERK (A,B) and by chemiluminesence for p38 (C,D). Activity levels were normalized to tubulin as a loading control. ERK activation was quantified as a percentage of basal activation in buffer-treated cells (0 min) pre-treated with DMSO or Compound E (panel B). In panel D p38 activation was quantified as O.D. values as detailed in Materials and Methods. ERK activity phenotypes are graphically represented in control fibroblasts compared with AD pre-disposed Trisomy 21 fibroblasts (B), and p38 activity phenotypes are graphically represented in control and PS-1 (M146L) AD fibroblasts (D). All graphs show mean±S.E. Statistical analysis was performed via t-test with **p-value<0.005; n = 3.

In the PS-1 AD fibroblasts, BK-induced ERK activation was significantly lower (2 fold) than that of the control fibroblasts at the very outset of BK stimulation. Given the known functional role of PS-1 as the catalytic core of the gamma-secretase complex generating the AD-related amyloid beta peptide [41], we defined the impact of a chemical inhibitor of gamma-secretase activity on early BK-mediated ERK activation. We tested the inhibitor Compound E at 1–10 nM concentrations decreasing gamma-secretase activity by 80–90% [42]. Normal control and PS-1 (M146L) AD human skin fibroblasts were pre-treated with Compound E (1 or 10 nM) or DMSO vehicle control overnight in complete culture media and then stimulated with 250 nM BK. In the presence of DMSO vehicle alone, BK-stimulated ERK activation was significantly decreased (2 fold) in PS-1 (M146L) AD fibroblasts relative to controls (Figure 8A–B). Compound E at 1 nM or 10 nM corrected the deficit in BK-stimulated ERK activation in the PS-1 (M146L) AD fibroblasts, significantly boosting BK-induced ERK activation by 2 fold, as compared to the vehicle only treatment. In contrast, Compound E had no effect on BK-mediated ERK activation in the normal control fibroblasts (Figure 8A–B) or in AD pre-disposed Trisomy 21 fibroblasts and the latters' age-matched controls (Figure 9A–B). PS-2 (N141I) AD fibroblasts treated with 10 nM Compound E also displayed no effect on BK-induced ERK activation (data not shown).

In additional experiments we probed the effect of Compound E on BK-mediated p38 activation (Figure 9C–D). Significant blunting of p38 activation was evident at 2 min of BK stimulation in PS-1 (M146L) fibroblasts relative to normal control fibroblasts. However, in cells treated with 10 nM Compound E the activation of p38 in the AD and normal fibroblasts was indistinguishable, suggesting that this gamma-secretase inhibitor corrects the aberrant signaling in both ERK and p38 pathways in the PS-1 (M1146L) fibroblasts.

### Hydrogen peroxide-induced oxidative stress evokes lagging ERK activation and enhanced JNK activation in AD fibroblasts

The diversified response to BK-mediated inflammatory stress in the various AD fibroblast lines, compared not only to normal age-matched controls but also to each other, prompted us to define the effects of oxidative stress on MAPK activation as well. Given the sustained BK pathway signaling particularly evident in PS-1 M146L AD fibroblasts, we compared the oxidative stress responses between this AD cell line and normal control fibroblasts, to determine whether the profiles of the two stresses, oxidative and BK-induced inflammatory, were the same or different. Human skin fibroblast lines were subjected to oxidative stress with H_2_O_2_ (250 µM) for 0–60 min, then re-fed with complete culture medium and monitored over a post-stress time window of 2–3 hr. Cell lysates collected in 1% SDS were immunoblotted for expression and activation of ERK, JNK and p38 and normalized to the loading control protein tubulin.

**Figure 10:**
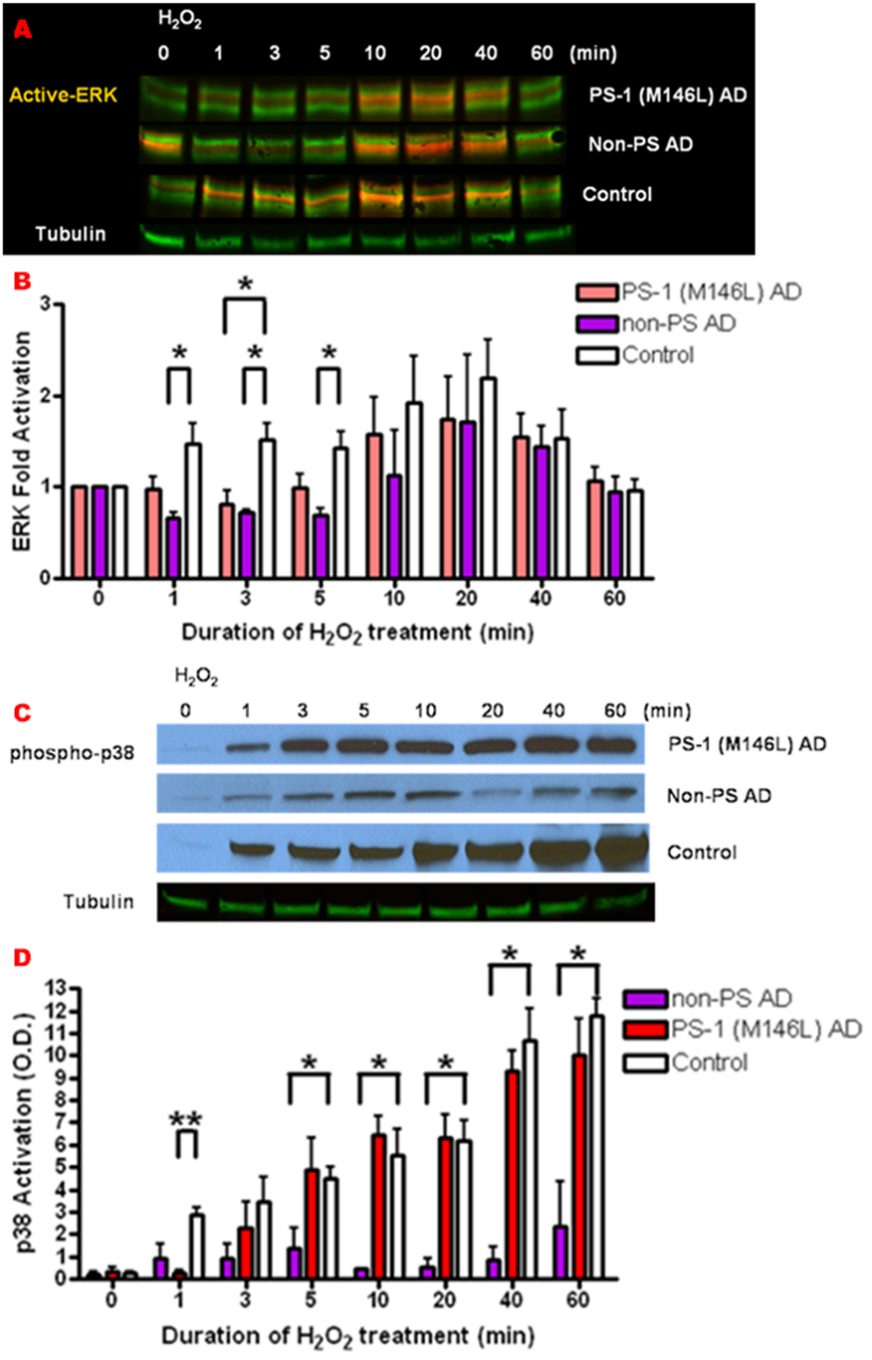
Hydrogen peroxide prompts lagging ERK and p38 activation in AD fibroblasts. PS-1 (M146L) and non-PS AD and normal control human skin fibroblasts were treated with 250 µM H_2_O_2_ for 0–60 min and immunoblotted for ERK and p38 as described in previous figures. ERK activation was digitally analyzed by Odyssey (A). ERK fold activation was quantified as a percentage of basal activation in buffer treated cells and normalized to Tubulin as loading control (B). Phospho-p38 levels were analyzed by ECL (C) and p38 activation was quantified as O.D. units normalized to tubulin as loading control (D). All graphs show mean±S.E. Statistical analysis was performed via t-test with *p-value<0.05 and **p<0.005; n = 4.

Normal control fibroblasts exhibited an immediate increase in ERK activation upon H_2_O_2_ treatment, which peaked at 10–20 min and terminated by 60 min and was significantly higher than the AD fibroblasts at up to 5 min (Figure 10A–B). In contrast, the PS-1 (M146L) AD fibroblasts exhibited a pronounced lag in ERK activation in the first 5 min of H_2_O_2_ exposure. ERK activation in the AD cells caught up to that of normal control fibroblasts by 10–20 min and decreased in parallel with control cells by 60 min. A striking difference from the case of BK inflammatory stress activating ERK was evident in the non-PS ApoE4 homozygote AD fibroblast line (Figure 10A–B). Where as this cell line had responded to BK equivalently to normal control fibroblasts [8] (Figure 6C–D), induction of oxidative stress prompted a temporally lagging ERK activation profile relative to control fibroblasts in the non-PS cell line that was comparable to the PS-1 (M146L) cells. In both of these AD cell lines, the decreased ERK activity during the lag period was statistically significant.

In contrast to the uniformly blunted oxidative stress-induced ERK activation in PS and non-PS AD vs. control fibroblasts, activation of the p38 MAPK module showed a differential activation profile in response to H_2_O_2_ depending on the origin of the AD (Figure 10C–D). At 1 min of H_2_O_2_ treatment, the control fibroblasts displayed significantly higher p-p38 levels (10 fold) than did PS-1 (M146L) AD fibroblasts. The PS-1 (M146L) AD fibroblasts displayed a lag at 1 min of H_2_O_2_-induced p38 activation but then by 3–5 min caught up to control fibroblasts for the remainder of the 1 hr time course of H_2_O_2_ treatment. p38 activation was still evident at 3 hr after termination of oxidative stress and re-feeding in both the PS-1 (M146L) AD and control cells (data not shown). As further evidence of the diversity of signaling profiles in fibroblasts from different AD origins, the non-PS AD fibroblasts exhibited a substantially diminished p38 activation in response to oxidative stress (Figure 10C–D). This p38 activation never attained the levels seen in either the PS-1 (M146L) or the control fibroblasts.

**Figure 11:**
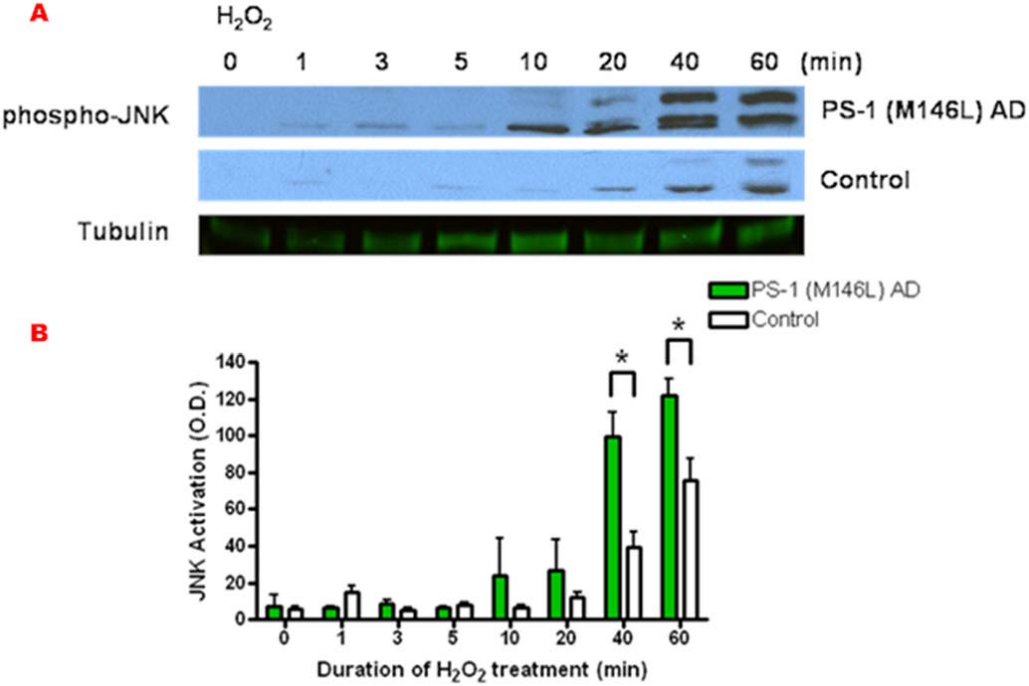
Hydrogen peroxide prompts enhanced JNK activation in PS-1 (M146L) AD fibroblasts. PS-1 (M146L) AD and normal control human skin fibroblasts were treated with 250 µM H_2_O_2_ for 0–60 min and immunoblotted for JNK activity as described in Figure 4. JNK activation was quantified as O.D. units normalized to tubulin as loading control (B). All graphs show mean±S.E. Statistical analysis was performed via t-test with *p-value<0.05; n = 4.

In marked contrast to the other two MAPK modules, H_2_O_2_-induced JNK activation displayed a significantly enhanced profile in the PS-1 M146L AD fibroblasts relative to controls. Initially both AD and control fibroblasts displayed a relative delay in the onset of JNK activation (Figure 11A–B) as compared with the swift onset of H_2_O_2_-induced ERK and p38 activation (Figure 10A–D). However, rather than a blunted response like that of ERK and p38, JNK in the PS-1 (M146L) AD fibroblasts achieved statistically significant 2-fold greater activation over that of control fibroblasts at 40–60 min of H_2_O_2_ treatment. The control fibroblasts did successfully activate JNK in response to H_2_O_2_, but at a comparatively lower magnitude. Thus H_2_O_2_-induced oxidative stress resulted in early lagging ERK and later enhanced JNK activation in the PS-1 (M146L) AD fibroblasts. We next compared how the downstream cellular response to oxidative stress is executed in the control versus PS-1 (M146L) fibroblasts.

### Oxidative stress is associated with enhanced apoptosis in PS-1 (M146L) AD fibroblasts

**Figure 12:**
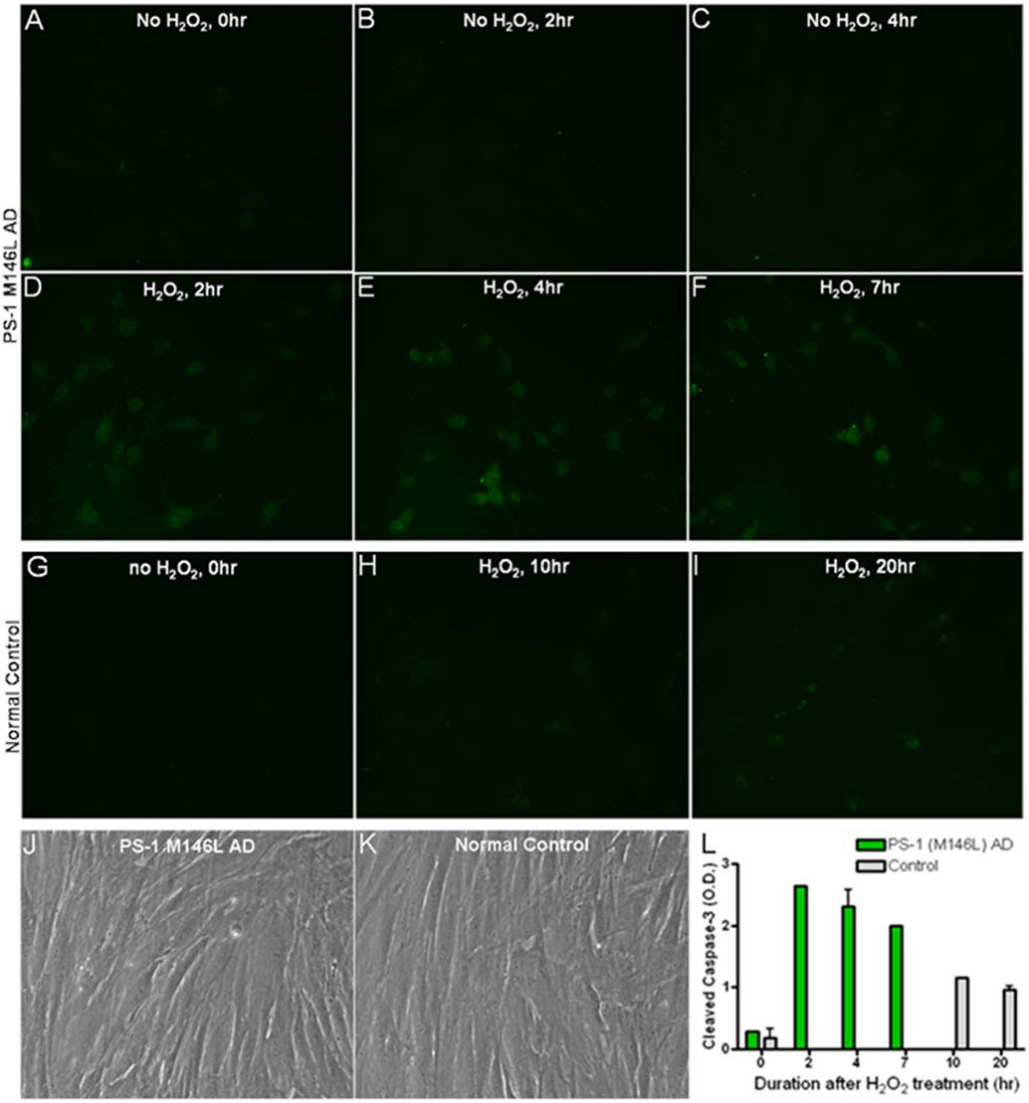
Hydrogen peroxide treatment results in heightened caspase-3 activation in PS-1 (M146L) AD fibroblasts. PS-1 (M146L) AD and control fibroblasts were treated with 500 µM H_2_O_2_ for 60 min and then re-fed with media for 0–20 hr to permit a post stress time window. Cells were methanol-fixed and analyzed by immunocytochemistry for cleaved caspase-3 with Alexa-488 labeled secondary antibody as described in Methods. Representative images of fields uniformly containing 35–40 cells were captured at time points designated (A–F). PS-1 (M146L) AD fibroblasts; (G–I) control fibroblasts. Representative phase contrast images of (J) control and (K) PS-1 (M146L) AD cells show comparable growth properties and cell densities at time of testing for oxidative stress responses. (L) Alexa 488 fluorescence intensity of cleaved caspase-3 immunostaining in PS-1 (M146L) fields A–F above and control fields G–I was quantitated via the MacBiophotonics Image J analysis program (www.macbiophotonics.ca/downloads.htm). Bar graph represents 2 independent experiments' mean and S.E.

Oxidative stress inducers are believed to participate in apoptosis, APP processing, Aβ secretion and τ phosphorylation [15]. Stress kinases such as the MAPKs both respond to oxidative stress, serving as reporters, and prompt downstream events resulting from the stress, such as apoptosis [9], [23], [24]. We therefore examined two hallmarks of cellular apoptosis: caspase-3 activation measured by immunofluorescent antibody recognition of its cleavage product [43], [44] and nuclear condensation detected by Hoechst 33258 staining [45]. Normal control and PS-1 (M146L) AD human skin fibroblasts were stimulated with 250–500 µM H_2_O_2_ for 0–60 min and then re-fed and monitored in the post stress time window for up to 24 hr. The cells were methanol fixed and stained for the apoptotic state markers. Both the normal control and PS-1 (M146L) fibroblasts displayed no caspase-3 activation within the initial 60 min of H_2_O_2_ treatment. However, subsequent to quenching of the oxidative stress by re-feeding the cells, the activation of caspase-3 showed a differential temporal profile in the PS-1 (M146L) AD fibroblasts as compared to the controls. H_2_O_2_ caused caspase-3 activation by 2 hr post stress in the PS-1 (M146L) AD fibroblasts, and the proportion of these cells exhibiting cleaved caspase-3 remained elevated at 7 hr post stress (Figure 12A–F). In contrast, the normal controls only began showing minimal caspase-3 activation by 20 hr post stress (Figure 12G–I). Phase contrast images of AD and control cells confirm that these cell lines had equivalent growth properties and were tested at equivalent cell densities (Figure 12J–K). Quantitation of the relative fluorescence intensity of caspase activation revealed a significant 8–10 fold enhancement of caspase-3 activity in the PS-1 (M146L) AD fibroblasts between 2 and 7 hr after oxidative stress (Figure 12L). The degree of caspase-3 activation attained in the normal control fibroblasts was approximately one third of that found in the AD fibroblasts and occurred much later, at 10–20 hr after oxidative stress.

**Figure 13:**
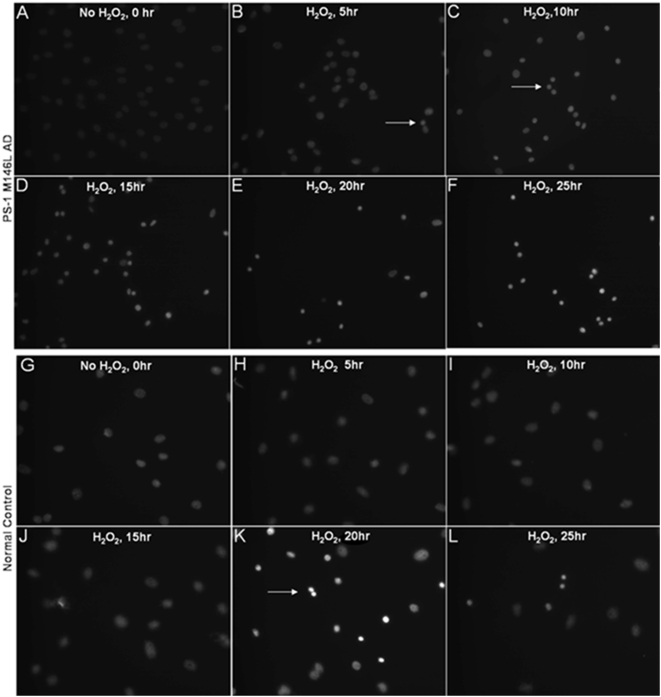
Hydrogen peroxide treatment results in enhanced nuclear condensation in PS-1 (M146L) AD fibroblasts. PS-1 (M146L) AD and control fibroblasts were treated with 500 µM H_2_O_2_ for 60 min and then re-fed with media for 0–25 hr. Cells were methanol-fixed and Hoechst stained as described in Methods to label nuclei and distinguish between normally sized nuclei versus condensed apoptotic nuclei. Hoechst staining is displayed in grey-scale for PS-1 (M146L) AD fibroblasts (A–F) and control fibroblasts (G–L). Representative captured images shown display signs of apoptosis with H_2_O_2_ treatment at designated time points.

**Figure 14:**
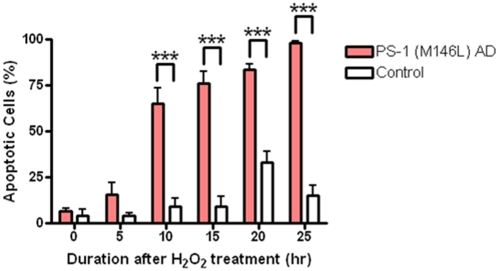
Hydrogen peroxide treatment results in an increased proportion of apoptotic nuclei in PS-1 (M146L) AD fibroblasts. PS-1 (M146L) AD and control fibroblasts treated with 500 µM H_2_O_2_ and re-fed with media for 0–25 hr were methanol-fixed, Hoechst stained, and the number of total and apoptotic nuclei quantitated. Graph shows percentage of condensed nuclei relative to total number of nuclei, mean±S.E. Statistical analysis was performed via t-test with ***p<0.0005; n = 3.

PS-1 (M146L) AD fibroblasts responded to oxidative stress with early nuclear condensation evident by Hoechst staining at 5–10 hr post stress (Figure 13A–F), whereas the control normal cells only exhibited nuclear condensation by 15–20 hr post stress (Figure 13G–L). The quantitation shown in Figure 14 demonstrates that the proportion of apoptotic nuclei arising after oxidative stress reached 60–95% of the cells in the culture during the post-stress time window from 10 hr up to 25 hr in the PS-1 (M146L) AD fibroblasts, whereas apoptotic nuclei in control fibroblasts reached at most 30% of the total cells at 20 hr after oxidative stress. Thus these AD fibroblasts exhibit a greater vulnerability to oxidative stress than do normal fibroblasts.

### A pro-survival function of JNK countering oxidative stress is lost in PS-1 (M146L) AD fibroblasts

**Figure 15:**
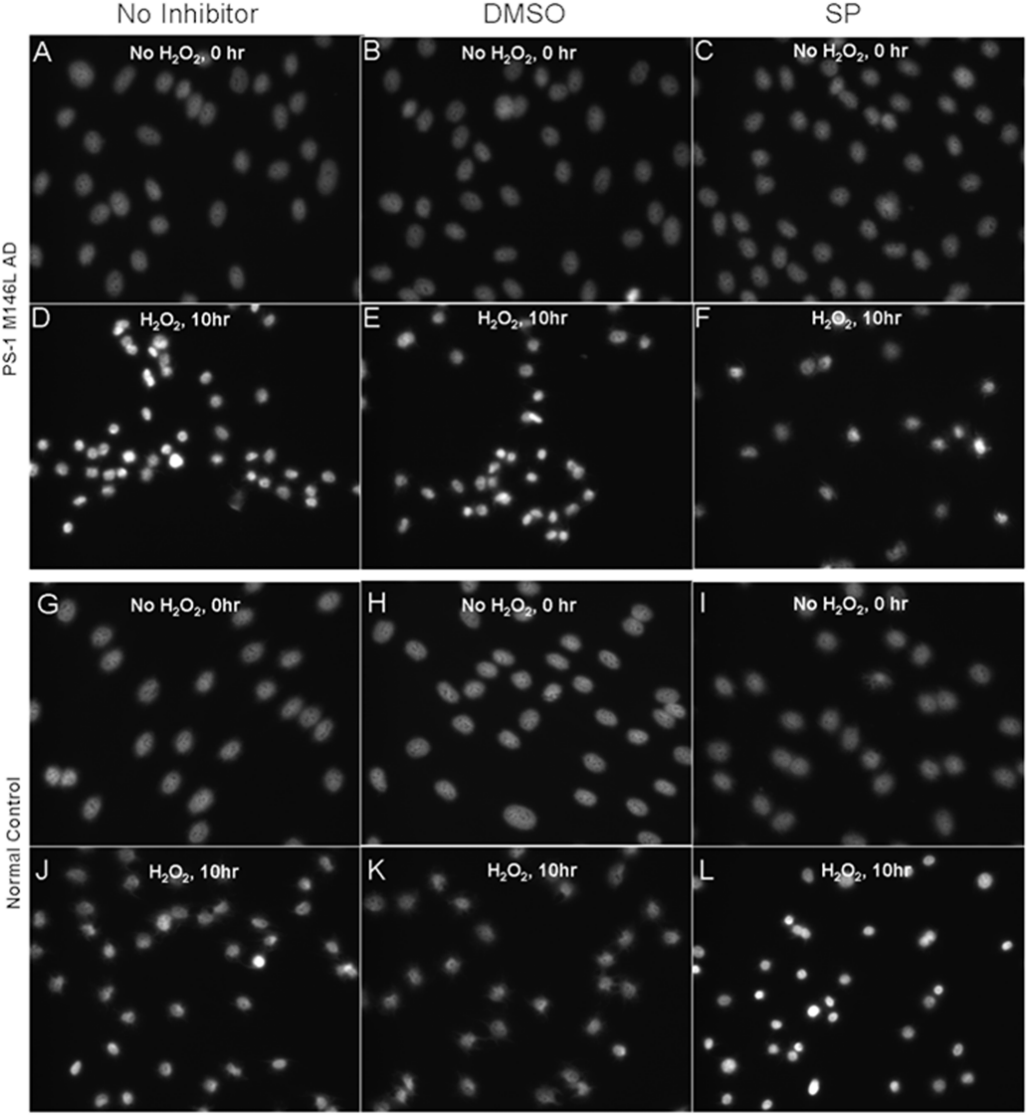
JNK inhibitor SP 600125 enhances oxidative stress apoptosis in control fibroblasts. PS-1 (M146L) AD and control fibroblasts were pre-treated with DMSO vehicle control or JNK inhibitor SP 600125 (25 µM) for 45 minutes in buffer. Oxidative stress was induced with 250 µM H_2_O_2_ for 60 min and cell layers were then re-fed with media plus vehicle control or JNK inhibitor for 0–10 hr. Cells were methanol-fixed and Hoechst stained to label nuclei and distinguish between normally sized nuclei versus condensed apoptotic nuclei. The No Inhibitor column (Panels A, D, G, and J) represents cells incubated in buffer without DMSO or SP 600125 but in the presence or absence of H_2_O_2_ during the initial 60 min incubation. Hoechst staining is displayed in grey for PS-1 (M146L) AD fibroblasts (A–F) and control fibroblasts (G–L). Representative captured images shown display signs of apoptosis with H_2_O_2_ treatment at designated time points.

**Figure 16:**
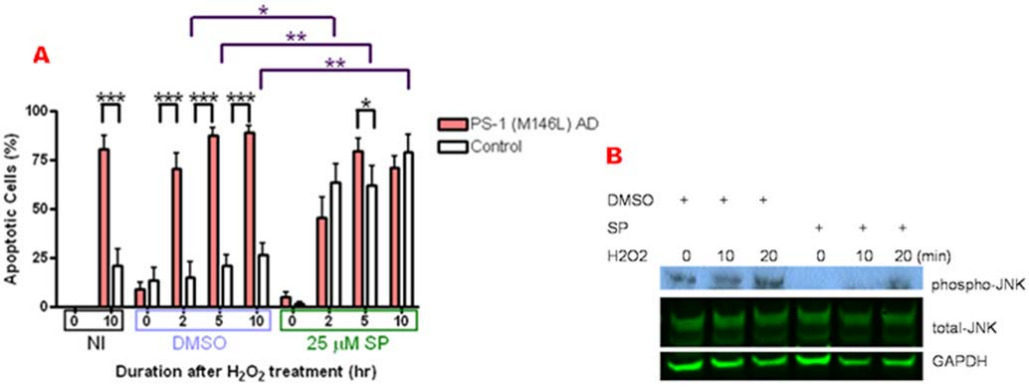
JNK promotes a survival function in normal fibroblasts that is lost in PS-1 (M146L) AD fibroblasts. PS-1 (M146L) AD and control fibroblasts were pre-treated with DMSO vehicle control or SP 600125 inhibitor in buffer, oxidatively stressed with 250 µM H_2_O_2_ for 60 min and re-fed with media for 0–10 hr, then methanol-fixed and Hoechst stained as previously described. (A) Quantitation of apoptosis due to H_2_O_2_. The No Inhibitor condition (NI) represents buffer without DMSO or SP 600125, with or without H_2_O_2_ during the 60 min incubation. The number of apoptotic nuclei was quantitated as a percentage of condensed nuclei to total nuclei per field. Graph shows mean±S.E. Statistical analysis was performed via t-test with *p-value<0.05; **p<0.005*** and p<0.0005; n = 3. Black * and *** brackets compare the percentage of apoptotic cells between AD and control fibroblast lines in the presence of DMSO or SP600125. Purple * and ** brackets compare the percentage of apoptotic cells within the control fibroblast line between the DMSO and SP600125 treatments. (B) Confirmation of SP600125 inhibition of JNK. PS-1 (M146L) AD fibroblasts pre-treated with DMSO or SP600125 for 45 min and then with 250 µM H_2_O_2_ for 0–20 min were immunoblotted and analyzed for phospho-JNK and total JNK by ECL and Odyssey respectively, normalized to GAPDH as a loading control.

We postulated that the enhanced magnitude of JNK activation in PS-1 (M146L) AD fibroblasts might be a potential mechanistic link to the enhanced oxidative stress-induced apoptosis in these cells relative to normal fibroblasts. Thus we pre-treated both cell types with the JNK inhibitor SP 600125 to test whether blocking JNK activation by oxidative stress would rescue the AD fibroblasts. However, the profile of brightly fluorescent condensed nuclei characteristic of apoptosis was equivalent in the PS-1 (M146L) fibroblasts in the absence or the presence of SP 600125 (Figure 15A–F). Surprisingly instead, inhibition of JNK by SP 600125 in the normal fibroblasts caused a striking enhancement of apoptosis evident at 10 hr after oxidative stress (Figure 15G–L). In the absence of JNK inhibition, the response of normal control fibroblasts after oxidative stress amounted to at most 30% apoptotic nuclei at both 250 µM (Figure 16A) and 500 µM (Figure 14) H_2_O_2_ by 10 hr after oxidative stress. With or without SP 600125 the PS-1 (M146L) AD fibroblasts approached 100% condensed nuclei. However, the proportion of normal cells exhibiting nuclear condensation after oxidative stress in the presence of >90% JNK inhibition by 25 µM SP 600125 (Figure 16B) became equivalent to that observed throughout in the PS-1 (M146L) fibroblasts (Figure 16A). Blockade of JNK did not significantly enhance apoptosis in normal control fibroblasts that were not subjected to oxidative stress, confirming the specificity of the JNK effect for the stress state. Thus human skin fibroblasts require an active JNK-mediated anti-apoptotic pathway for protection against the deleterious effects of oxidative stress. This anti-apoptotic mechanism is no longer functional in PS-1 (M146L) fibroblasts despite their 2-fold greater elevation of active JNK upon oxidative stress relative to normal cells.

## Discussion

Dysfunction of cellular signaling represents a prominently emerging focus in the pathogenesis and pathobiology of Alzheimer's Disease (AD). Signaling within and among cells and tissues has been offered as a prominent platform for ascertaining the risk of developing Alzheimer's disease [11], [46], [47], [48], [49], [50]. We therefore tested whether stimuli characteristic of inflammatory stress and oxidative stress, known risk factors *in vivo* for the development of AD, each resulted in the same or in unique differential responses of signaling cascades. We employed skin fibroblasts from normal individuals as well as fibroblasts from persons with well-defined highly penetrant genetic forms of AD. Inflammatory and oxidative stress induction with BK and H_2_O_2_ respectively, significantly altered the activity of all three MAPK modules, pinpointing the ERK cascade as a significant stress responder in addition to p38 and JNK. However the activity phenotypes of each MAPK module in AD cells were intriguingly distinct based not only on the characteristics of the stress inducer but also on the nature of the underlying disease-causing mutation or risk factor. Our findings challenge the concept of a unitary signaling test or biomarker for AD risk, and point instead to an emerging complexity in molecular profiles based in hierarchies of signaling pathways that are subject to dysregulation among varying origins of AD.

Our studies began from the vantage point of defining aberrant intracellular signal transduction that may reflect a loss of function, a redirection of key pathways, and/or a concurrent toxic gain of function, all taking place in AD vulnerable cells [51]. The results presented here demonstrate that complex profiles of losses and gains of functions indeed comprise the signaling landscape of cells derived from AD of different molecular origins. The components of such a balance sheet in AD fibroblasts include gains in the presence and lifetime of aberrantly functioning BKB2 receptors that promote AD-relevant *tau* phosphorylation [8] and a gain in the absolute level of JNK activation that is concurrent with loss of a key protective role for JNK in countering oxidative stress. Other losses are evident in the time courses and/or ultimately attained levels of MAPK activation in the ERK and p38 modules that are distributed differentially among different molecular bases for AD. Thus diversity is the primary distinguishing feature of altered signal transduction in accessible peripheral tissue cells in AD.

The altered PKC-dependent signal transduction pathway we defined in skin fibroblasts from AD patients, yielding BKB2R modulated by phosphorylation [8], [13], [14], is discernible in Trisomy 21 fibroblasts decades before the characteristic age of onset of symptomatic AD. A common element of this molecular profile appearing in all presenilin and non-presenilin-based genetic forms of AD risk we tested is the initial BK-induced BKB2R Tyr phosphorylation itself, positioned early in the BKB2R signaling cascade [8], [12], [13], [14]. Beyond this initial step, our present studies now reveal multiple points of divergence in the downstream signaling cascades in AD cells of differing genetic origins.

A primary divergence in signaling pathways links the cellular half-life of the phosphorylated BKB2R generated by PKC stimulation to respective sites of presenilin mutations all located in the N-terminal domain of the molecule (Figure 1B). PS-1 M146L mutated in the second transmembrane domain exhibits a prolonged phosphorylated BKB2R signal whereas that of both PS-1 A246E in the sixth transmembrane domain and nearby PS-1 L286V in the hydrophobic region of the cytosolic loop domain are relatively transient. Mutations in the N-terminal portion of the PS-1 molecule appear to promote cytotoxic effects by a common mechanism involving nitric oxide generation [35]. The latter second messenger is also a known downstream target of the BKB2R [52]. The site-specific differences in BKB2R signal strength we observe parallel the degree to which the PS-1 mutation is an aggressive one, reflected in both an earlier age of onset of the disease and in secretion of Aβ_1–42_ into the culture medium by AD skin fibroblasts and PS-1-expressing cell lines. PS-1 M146L AD has one of the earlier ages of onset among PS-1 mutations, occurring below age 40, and skin fibroblasts from such individuals elaborate a 9-fold greater amount of Aβ peptide than fibroblasts from normal individuals. This compares with PS-1 A246E and L286V mutations whose age of AD onset is generally later than 40 and whose fibroblasts secrete 2-3-fold more Aβ_1–42_ than normals [53], [54].

Although mutations in the presenilin family of proteins can be causative for AD, the links between presenilin functions and signal transduction events in the tripartite MAPK pathways remain to be fully explained. The skin fibroblasts employed here express PS-1 and PS-2 in their normal or mutated forms at natural endogenous levels, obviating the issue of quantitative effects frequently present in other types of expression systems. Under these circumstances, when we tested BKB2R responses to the pathophysiologic levels of 25–250 nM BK, the phenotype of each active MAPK module showed intriguingly distinct properties based on the nature and characteristics of both the origin of the AD and the particular stress induced.

PS-1 fibroblasts with Familial AD mutations at M146L, A246E and L286V all selectively demonstrated a lagging and quantitatively decreased BK-induced ERK activation, as compared to age-matched normal control fibroblasts. In contrast, the PS-2 N141I, non-PS AD and Trisomy 21 fibroblasts displayed BK-induced ERK activity equivalent in duration and magnitude to that of normal control cells. The PS-2 N141I Familial AD mutation represents the homologous mutation to that of PS-1 M146L [8], [55], and yet the BK-induced ERK phenotypes are surprisingly completely different. We hypothesize that this may reflect differential PS-1 versus PS-2 roles in the signal transduction cascades proceeding onward to ERK activation.

The differential lag in BK-mediated ERK signaling we observed in PS-1 mutant versus PS-2 mutant and other AD fibroblasts could shed light on the function of PS-1 as the key catalytic component of gamma-secretase activity. This multi-protein complex, composed of PS-1, PS-2, PEN2, Aph1 and nicastrin (NCT), cleaves numerous membrane-embedded substrates including APP and Notch [38], [56], [57], [58]. Some familial AD PS-1 mutations have been suggested to be indicative of a gain of function of gamma-secretase [56], [59], [60], [61], [62]. A PS-1-selective role here is bolstered by our finding that pre-treatment with the gamma-secretase inhibitor Compound E preceeding BK stimulation in the PS-1 (M146L) AD fibroblasts corrected BK-mediated ERK activity to the levels observed in normal control fibroblasts. Compound E had neither an effect on BK-induced ERK activation in Trisomy 21 and control fibroblasts, nor on BK-stimulated ERK in PS-2 (N141I) AD fibroblasts. Hence the diminished BK-induced PS-1 ERK phenotype correctable by gamma-secretase inhibitor Compound E may reflect functional differences in the catalytic core of the gamma-secretase complex generating aspartyl protease activity unique to PS-1 as opposed to PS-2 [38], [41], [56], [57], [63], [64], or alternatively in ER Ca^2+^ metabolism [41], [65], [66]. These respective PS roles may or may not be independent of each other [65]. PS-1 mutation appears to exert a greater severity at several levels of BK signaling than the homologous PS-2 mutation, from BKB2R modulation to ERK activation. PS-1 FAD mutations are also more prevalent than those of PS-2 FAD, underscoring the role of PS-1 in gamma-secretase related AD pathology [41], [64], [67], [68].

The responses of the p38 module to activation by BK inflammatory stress both paralleled and diverged from the ERK responses, in fibroblasts of differing AD origins. The PS-2 mutant cells resembled PS-1 mutants in regard to p38 and normal cells in regard to ERK, while Trisomy 21 cells exhibited both BK-dependent p38 activation and ERK activation that was equivalent to normal fibroblasts despite showing other avenues of signal transduction that are like presenilin AD fibroblasts [8]. The functional divergences observed between ERK and p38 may relate to upstream steps in this overall pathway differentially linked to presenilin functions that remain to be defined.

The genetic background of PS-1 FAD mutations has been proposed to correlate with increased vulnerability to oxidative stress and apoptosis [62], [69], [70], possibly due to aberrant PS-1 function in maintaining ER calcium homeostasis [65], [67], [69]. Since inflammatory stress yielded a diminished BK-induced activation of all three MAPK modules in PS-1 M146L, we initially assumed that oxidative stress would likely have a similar effect. Oxidative stress has been consistently viewed as an early event in the development and pathogenesis of AD and the consequent generation of reactive oxygen species (ROS) is particularly toxic to the brain [71], [72], [73]. In our AD fibroblast model the highly penetrant single gene disorder of familial AD based on the PS-1 M146L mutation exerts a strong effect on fibroblast signaling to severely disrupt cellular signaling homeostasis in oxidative stress as well as inflammatory stress, but with a different molecular signature from that of BK. In the PS-1 M146L AD human skin fibroblasts whose aberrant BK signaling was the strongest, oxidative stress induced a massive enhancement of JNK activation concomitant with lagging p38 and ERK activation. A lagging MAPK activation phenotype upon oxidative stress was also evident for p38 in the non-presenilin based familial AD fibroblast line, where p38 activation across the entire hour of oxidative stress was even more strikingly diminished. Thus in these non-presenilin AD cells oxidative stress responses present a striking contrast to the inflammatory stress of BK-stimulated ERK, whose profile was equivalent to that of normal cells.

Initial differential activation of MAPK pathways by oxidative stress was followed by a heightened and earlier degree of apoptosis in the PS-1 M146L fibroblasts relative to normal fibroblasts. Effectively all of the AD fibroblasts underwent programmed cell death beginning within 2 hours after oxidative stress, whereas at most 30% of the normal control fibroblasts incurred this fate and then only beyond 10–20 hours after oxidative stress. The JNK inhibitor SP600125 failed to block the oxidative stress-induced enhancement of apoptosis in the PS-1 (M146L) AD cells, indicating that the latter stress response does not arise solely from exaggerated activation of JNK. Rather, the SP600125 conversion of the normal cells' apoptotic profile to one approximating that of PS-1 (M146L) cells underscores a protective role for JNK in countering oxidative stress to human skin fibroblasts. JNK is more prominently known as a pro-apoptotic than an anti-apoptotic signaling pathway [74], [75], [76], [77], [78], [79], so that its key survival function for normal human skin fibroblasts in the face of oxidative stress, and the disappearance of this protection in PS-1 (M146L) fibroblasts, represents a novel finding. The enhanced JNK activation seen in the AD cells, rather than serving as a pathogenetic mechanism, may reflect a cellular reporting system for the existence of an environmental stress and/or a compensatory response that nonetheless fails to accomplish survival of the AD cells. Further studies are required to determine how MAPK or other signaling cascades may be involved in propagating the toxic effects of H_2_O_2_ [80], [81], for example by cross-talk mechanisms affecting intracellular redox balance [24]. The lag in H_2_O_2_-induced ERK and p38 activation in the AD fibroblasts merits future studies to determine if this delay may compromise mobilization of a protective response to oxidative stress linked to JNK activation arising later in the course of oxidative stress.

Our diverse findings with BK-induced inflammatory stress and oxidative stress thus return to the question of what type(s) of testing for AD risk can achieve not only sufficient specificity and sensitivity but also practicability to be a realistic option for all individuals among the general population [46], [47], [48], [50]. Familial AD due to highly penetrant mutations in presenilins or amyloid precursor protein is a rare condition detected by history plus genetic analysis for outright disease-causing mutations or haplotype associations with other suspects [82], [83], [84], [85]. Cost and technical issues tend to constrain AD research and management based in invasive procedures to high-risk individuals [49], [86], [87], [88]. The more prevalent sporadic AD represents the pre-eminent development target for both risk assessment and therapeutic strategies. This AD [89], [90] manifests as a complex multigenic disorder, attributed to the conglomerate effects of small variations in many biochemical parameters, that may have not only genetic but epigenetic and environmental bases [48], [91], [92], [93]. These contributors in sporadic AD are believed to collectively replicate the phenotype of strong monogenic causes of AD whose molecular signatures we have established in the MAPK and BK signaling cascades.

Our results emphasize the need to consider, in probing AD at any level, a broad spectrum of inflammatory, oxidative, and other stressors as well as a broad view of the intracellular signaling landscape responding to these stresses. A focus on a single MAPK species or stressor could miss molecular signatures that may be informative about cellular environments generated by PS-2 mutations as well as the proteomic manifestations of Trisomy 21 that pertain to AD pathogenesis. In current clinical assessment of AD pathogenesis combined biomarkers provide added value [94], [95], and the significance of a similar approach at the cellular and molecular level in AD models expressing endogenous levels of relevant pathogenic candidates is underscored by our findings.

Our molecular profiling of signal transduction cascades in AD cellular models indicates that the disease processes at the cellular level even in strongly monogenic AD are complex and multifactorial. AD human skin fibroblast models may offer a unique opportunity for uncovering AD pathogenetic mechanisms if recently-developed isolation procedures for induced pluripotent stem cells (iPSC's) from fibroblast populations can be deployed to generate AD-specific human neuronal populations [96], [97]. Dysregulation in both phosphorylation- and protease-based signaling cascades revealed in this newly-emerging type of cellular environment could translate insights from reprogrammed human neurons expressing strong single-gene-causative AD into the spectrum of individual small differences that may contribute to sporadic AD. Furthermore, development of such a system may suggest novel therapeutic targets to combat this disorder far ahead of its earliest clinical manifestations. Therapeutic interventions for other complex multigenic disorders such as diabetes, hypertension, and cardiovascular disease are not confined to a single modality. Thus therapeutic strategies addressing the nature of AD in any form are likely to themselves be complex rather than monolithic.

## Materials and Methods

### Reagents

Material [Source]: DMSO, Hydrogen Peroxide, Bradykinin triacetate salt, SP 600125 [Sigma Aldrich], Compound E [Alexis Biochemicals], Fetal bovine serum [Hyclone], 8% or 4–20% Precise Protein Gels [PIERCE], Hoechst 33258 [Sigma Aldrich], Nestle Instant Powdered Milk, Vectastain ABC kit, Biotinylated 2° antibodies [Vector Laboratories], Trans-Blot Transfer Medium [Bio-Rad], 26 gauge Precision Glide Needle [Becton Dickinson; FISHER], Restore™ Western blot stripping buffer [PIERCE], Tween-20 [0.1%], Odyssey Blocking Buffer [Li-Cor], X-ray film [Midwest Scientific], Super HT Mini PAP Pen [RPI], Micro BCA Protein Assay Kit [PIERCE], Super signal West Pico Chemiluminescence Substrate [PIERCE], Phosphatase Arrest 1 [GBiosciences], AD and age-matched normal control human skin fibroblasts (see Table 1) [Coriell Institute].

### Cell culture conditions

#### AD and normal control human skin fibroblasts

Optimal culture conditions at 37°C in air containing 5% CO_2_ involved 4 day feeding cycles with media containing 90% v/v alpha-MEM, and 10% v/v fetal calf serum (Hyclone). Passaging of confluent cells cultured in T75 (Midwest Scientific) flasks required washes (2×) with Dulbecco's Phosphate-Buffered Saline (PBS) and 5 min treatment with 0.05% trypsin/0.54 mM (0.02%) EDTA as described previously [8]. Trypsinized cells were distributed for experiments into 6-well plates (Nunc), single-chamber slide flasks (Nunc) or T12.5 stock flasks (Falcon).

#### Oxidative stress induction

AD and control fibroblast lines (Table 1) were cultured to confluence in 3 mls media/T12.5 flask or slide flask. After washing twice with pH 7.5 Hepes-buffered Hank's balanced salt solution (HHBSS), the cells were treated with 250–500 µM H_2_O_2_ in human skin fibroblasts in phenol red-free HHBSS buffer for 1 hr at 37°C. Removal of the buffer and refeeding with complete medium for 1–25 hrs quenched any remaining oxidant species. The cells were either methanol fixed for immunofluorescence studies or cell lysates were harvested in 1% SDS detergent containing phosphatase inhibitors for immunoblotting studies.

#### Treatment with SP 600125

AD and control fibroblast lines were first washed with HHBSS (2×) and then pre-treated for 45 minutes with DMSO vehicle control or SP 600125 (25 µM) in HHBSS. Subsequently the oxidative stress induction was followed as described above. The entire time frame of inhibitor pretreatment, oxidative stress induction and the methanol fixation or detergent lysis of cells was performed in the dark due to light sensitivity of the inhibitor. Vehicle control and inhibitor were present in the buffer or media throughout the experimental time frame.

#### Inflammatory stress induction

AD and control fibroblast lines (Table 1) were treated with 25 or 250 nM BK for 0–30 minutes at 37°C. Detergent harvested cell lysates were immunoblotted with total and active phosphoepitope specific antibodies (Table 2) against each of the three MAPKs to determine their respective bulk protein expression and activity profiles upon BK stimulation.

**Table 2:** Immunoreagents.

Candidate protein	Manufacturer	Species	Dilution	Antibody	Application
Total-ERK	Cell Signaling	Rabbit	1∶1000	1°	WB
Phospho-ERK	Cell Signaling	Mouse	1∶1000	1°	WB
Total-JNK	Cell Signaling	Rabbit	1∶1000	1°	WB
Phospho-JNK	Stressgen	Mouse	1∶1000	1°	WB
Total-p38	Cell Signaling	Rabbit	1∶1000	1°	WB
Phospho-p38	Cell Signaling	Mouse	1∶1000	1°	WB
Pan α/βTubulin	Cell Signaling	Rabbit	1∶2000	1°	WB
Cleaved Caspase-3	Cell Signaling	Rabbit	1∶50	1°	IF
Alexa 488	Molecular Probes	Rabbit	1∶600	2°	IF
Alexa 594	Molecular Probes	Rabbit	1∶600	2°	IF
IRDye 800CW	Li-Cor (Odyssey)	Rabbit	1∶15,000	2°	WB
Alexa Fluor 680	Invitrogen (Odyssey)	Mouse	1∶15,000	2°	WB

WB = Western blotting, IF = Immunofluorescence.

#### Treatment with Compound E

AD and control fibroblast lines were pre-treated with DMSO vehicle control or Compound E (1 or 10 nM) overnight by adding the compounds to the complete serum-containing medium already surrounding the cells. After 24 hours, the inflammatory stress induction was followed as described above with washes and experimental treatment with BK in HHBSS buffer. The vehicle control and inhibitor were present in the buffer or media throughout the experimental time frame.

#### Immunoblotting and quantitation

Cell lysates collected in 1% SDS detergent were sheared with 26 gauge needles and protein was estimated with the Micro BCA Protein Assay Kit. Samples containing 30 µg total protein per lane were electrophoresed on pre-cast polyacrylamide Tris-HEPES-SDS gels (PIERCE) and transferred onto Nitro-Cellulose membranes (Bio-Rad). Subsequently, immunoblotting with the antibodies listed in Table 2 and individual or tandem application of ECL (enhanced chemiluminescence) and Odyssey infrared dual color imaging was used to determine protein expression and activity profiles of the MAPKs, in some cases multiple MAPK species on the same western blot.

For ECL analysis, blotted membranes were blocked for 1 hr at room temperature with freshly prepared non-fat dry milk (Nestle Instant 10% w/v) in Tris-Buffered Saline- Tween (TBS-T) blocking buffer and incubated with the primary antibody in blocking buffer or TBS-T overnight at 4°C. Washes (3×) with TBS-T were followed by incubation with biotinylated secondary antibodies (Vector Lab) for 1 hr at room temperature. The blots were washed (3×) and incubated with ABC amplification substrate, prepared 30 min before use. After final washes (3×) the blot was incubated with Pico ECL substrate (Pierce) for 5 min in the dark and exposed to X-ray film. The protein bands on the developed film were quantified by ImageJ (NIH: http://rsb.info.nih.gov/ij/). The activity levels of p38 and JNK were calculated by dividing the intensity of phospho-p38 or phospho-JNK by that of loading control proteins tubulin, actin, or GAPDH, chosen for the best resolution from the kinase bands of interest on the basis of M_r_. Relative activity levels were expressed as the relative fold activation of p38 and JNK observed in BK- or H_2_O_2_-stimulated cells compared to that of basal unstimulated cells (set at 1-fold). Because of the low expression levels of p38 and JNK in these cells, in some experiments the basal unstimulated activity was present at trace levels rendering quantitation difficult, so that calculating relative fold activation yielded excessively wide variation in stimulated values. In these cases relative activation was expressed in terms of optical density (O.D.) units alone. The optical densities were corrected for non-specific background on the exposed blots and normalized to loading controls as outlined above.

For Odyssey analysis either the above blot previously analyzed by ECL or a *de novo* blot was incubated with Odyssey Blocking Buffer (ODBB) for 1 hr at room temperature and then probed with primary antibodies in ODBB plus Tween-20 (0.1%) overnight at 4°C. Washes (3×) with Phosphate-Buffered Saline- Tween (PBS-T) were followed by incubation with green or red infrared dye-conjugated secondary antibodies (Table 2) for 1 hr at room temperature under dark conditions. Washes with PBS-T (3×) and a final wash in PBS without Tween plus storage in PBS protected from light enabled optimal storage of blots at 4°C and quantification of bands using the Odyssey Infra-red Imaging System. The activity levels of ERK were calculated by dividing the intensity of phospho-ERK levels to that of regular total ERK protein levels and normalizing that to loading control protein tubulin. Total protein content of p38 and JNK from blots previously analyzed for kinase activities by could thus be determined by the tandem ECL/Odyssey strategy and corrected for loading controls chosen on the basis of optimal band resolution as above. Total cellular levels of ERK, JNK, and p38 proteins did not change significantly over the time courses of up to 1 hour incubation with BK or H_2_O_2_.

#### Immunofluorescence (IF) and nuclear condensation

Human skin fibroblasts cultured in single chamber slide flasks (Nunc) following experimental or control treatments were fixed for 1 min in ice-cold methanol and stored in HHBSS at 4°C. For immunostaining, the HHBSS was aspirated and cell layers were dried at room temperature. Replicate hydrophobic rings were created with a PAP pen (RPI) on the cell layers for each experimental condition. The rings were either stored in HHBSS or blocked with IF blocking buffer (0.05 g BSA, 0.01 g Casein, 5 mLs HHBSS) at room temperature for 1 hr. After aspirating the blocking buffer, the primary antibody prepared in blocking buffer was applied (20 µL/ring) and incubated overnight at 4°C. Washes (3×) with HHBSS (20 µL/ring) were followed by incubation with Alexa 488 conjugated light sensitive secondary antibody prepared in blocking buffer at 4°C (Table 2). Incubation of secondary antibody at room temperature for 1 hr in the dark was followed by washes (3×) and storage with HHBSS (20 µL/ring) at 4°C. Hoechst staining to detect apoptotic nuclear condensation was carried out after aspirating HHBSS from each ring. Hoechst 33258 (0.5 µg/mL) was added (30 µL/ring) for 10 min at room temperature and protected from light. Washes (2×) were followed by storage in 20 µL HHBSS and storage 4°C. Immunofluorescence and Hoechst-stained images were captured and quantitated on an inverted microscope Nikon Eclipse TE2000-U via an Optronics Magnafire 2.0 digital imaging system using its associated AnalySIS software or alternatively the MacBiophotonics version of ImageJ (see Figure 12 legend).

## References

[1] Haddad J (2004). Mitogen-activated protein kinases and the evolution of Alzheimer's: a revolutionary neurogenetic axis for therapeutic intervention?. Progress in Neurobiology.

[2] Uryu K, Laurer H, McIntosh T, Pratico D, Martinez D (2002). Repetitive mild brain trauma accelerates Abeta deposition, lipid peroxidation, and cognitive impairment in a transfenic mouse model of Alzheimer amyloidosis.. Journal of Neuroscience.

[3] Janicki S, Stabler SM, Monteiro MJ (2000). Familial Alzheimer's disease presenilin-1 mutants potentiate cell cycle arrest.. Neurobiology of Aging.

[4] Marchesi V (2005). An alternative interpretation of the amyloid Abeta hypothesis with regard to the pathogenesis of Alzheimer's disease.. PNAS.

[5] Mattson M (2004). Pathways towards and away from Alzheimer's disease.. Nature.

[6] McGowan E, Pickford F, Kim J, Onstead L, Eriksen J (2005). Abeta42 is essential for parenchymal and vascular amyloid deposition in mice..

[7] Butterfield D, Castegna A, Pocernich CB, Drake J, Scapagnini G (2002). Nutritional approaches to combat oxidative stress in Alzheimer's disease.. Journal of Nutritional Biochemistry.

[8] Jong Y, Ford SR, Seehra K, Malave VB, Baengizer NL (2003). Alzheimer's disease skin fibroblasts selectively express a bradykinin signaling pathway mediating tau protein Ser phosphorylation.. The FASEB Journal.

[9] Chong Z, Li F, Maiese K (2005). Oxidative stress in the brain: Novel cellular targets that govern survival during neurodegenerative disease.. Progress in Neurobiology.

[10] Fowler CJ, Cowburn RF, Garlind A, Winblad B, O'Neill C (1995). Disturbances in signal transduction mechanisms in Alzheimer's disease.. Mol Cell Biochem.

[11] Zhao W, Ravindranath L, Mohamed AS, Zohar O, Chen GH (2002). MAP kinase signaling cascade dysfunction specific to Alzheimer's disease in fibroblasts.. Neurobiology of Disease.

[12] Jong YJ, Dalemar LR, Wilhelm B, Baenziger NL (1996). Human lung fibroblasts express multiple means for enhanced activity of bradykinin receptor pathways.. Immunopharmacology.

[13] Jong YJ, Dalemar LR, Wilhelm B, Baenziger NL (1993). Human bradykinin B2 receptors isolated by receptor-specific monoclonal antibodies are tyrosine phosphorylated.. Proc Natl Acad Sci U S A.

[14] Jong YJ, Dalemar LR, Seehra K, Baenziger NL (2002). Bradykinin receptor modulation in cellular models of aging and Alzheimer's disease.. Int Immunopharmacol.

[15] Olivieri G, Baysang G, Meier F, Muller-Spahn F, Stahelin HB (2001). N-Acetyl-L-cysteine protects SHSY5Y neuroblastoma cells from oxidative stress and cell cytotoxicity: effects on beta-amyloid secretion and tau phosphorylation.. Journal of Neurochemistry.

[16] Gibson G (2002). Causes and consequences of oxidative stress in Alzheimer's Disease.. Free Radical Biology & Medicine.

[17] Pratico D (2005). Peripheral biomarkers of oxidative damage in Alzheimer's disease: the road ahead.. Neurobiology of Aging.

[18] Smith M, Hirai K, Hsiao K, Miguel, Pappolla MA, Harris PLR (1998). Amyloid-beta deposition in Alzheimer transgenic mice is associated with oxidative stress.. Journal of Neurochemistry.

[19] Velez-Pardo C, Ospina GG, Jimenez del Rio M (2002). Abeta[25–35] peptide and iron promote apoptosis in lymphocytes by an oxidative stress mechanism: invovlement of H2O2, Caspase-3, NF-kappaB, p53 and c-Jun.. Neuro Toxicology.

[20] Behl C, Davis JB, Lesley R, Schubert D (1994). Hydrogen peroxide mediates amyloid beta protein toxicity.. Cell.

[21] Zhu X, Raina AK, Perry G, Smith MA (2004). Alzheimer's disease: the two-hit hypothesis.. Neurology.

[22] Zhu X, Raina AK, Lee H, Casadesus G, Smith MA, Perry G (2004). Oxidative stress signalling in Alzheimer's disease.. Brain Research.

[23] Yoshizumi M, Abe J, Haendeler J, Huang Q, Berk BC (2000). Src and Cas mediate JNK activation but not ERK1/2 and p38 kinases by reactive oxygen species.. The Journal of Biological Chemistry.

[24] Matsuzawa A, Ichijo H (2005). Stress-responsive protein kinases in redox-regulated apoptosis signaling.. Antioxidants and Redox Signaling.

[25] Hayashi R, Yamashita N, Matsui S, Fujita T, Araya J (2000). Bradykinin stimulates IL-6 and IL-8 production by human lung fibroblasts through ERK- and p38 MAPK-dependent mechanisms.. Eur Respir J.

[26] Liu B, Yu J, Taylor L, Zhou X, Polgar P (2006). Microarray and phosphokinase screenings leading to studies on ERK and JNK regulation of connective tissue growth factor expression by angiotensin II 1a and bradykinin B2 receptors in Rat1 fibroblasts.. J Cell Biochem.

[27] Liu J, Lin A (2005). Role of JNK activation in apoptosis: A double-edged sword.. Cell Reserach.

[28] Matsuzawa A, Nishitoh H, Tobiume K, Takeda K, Ichijo H (2002). Physiological roles of Ask-1 mediated signal transduction in oxidative sress- and endoplasmic reticulum stress-induced apoptosis: Advanced findings from Ask-1 knockout mice.. Antioxidants and Redox Signaling.

[29] Zhao WQ, Feng C, Alkon DL (2003). Impairment of phosphatase 2A contributes to the prolonged MAP kinase phosphorylation in Alzheimer's disease fibroblasts.. Neurobiol Dis.

[30] Zhu X, Raina AK, Rottkamp CA, Aliev G, Perry G (2001). Activation and redistribution of c-jun N-terminal kinase/stress activated protein kinase in degenerating neurons in Alzheimer's disease.. J Neurochem.

[31] Augustinack JC, Schneider A, Mandelkow EM, Hyman BT (2002). Specific tau phosphorylation sites correlate with severity of neuronal cytopathology in Alzheimer's disease.. Acta Neuropathol.

[32] Bartov O, Sultana R, Butterfield DA, Atlas D (2006). Low molecular weight thiol amides attenuate MAPK activity and protect primary neurons from Abeta(1–42) toxicity.. Brain Res.

[33] De Strooper B (2007). Loss-of-function presenilin mutations in Alzheimer disease. Talking Point on the role of presenilin mutations in Alzheimer disease.. EMBO Rep.

[34] Hardy J (2007). Putting presenilins centre stage. Introduction to the Talking Point on the role of presenilin mutations in Alzheimer disease.. EMBO Rep.

[35] Hashimoto Y, Tsukamoto E, Niikura T, Yamagishi Y, Ishizaka M (2004). Amino- and carboxyl-terminal mutants of presenilin 1 cause neuronal cell death through distinct toxic mechanisms: Study of 27 different presenilin 1 mutants.. J Neurosci Res.

[36] Wolfe MS (2007). When loss is gain: reduced presenilin proteolytic function leads to increased Abeta42/Abeta40. Talking Point on the role of presenilin mutations in Alzheimer disease.. EMBO Rep.

[37] Zatti G, Burgo A, Giacomello M, Barbiero L, Ghidoni R (2006). Presenilin mutations linked to familial Alzheimer's disease reduce endoplasmic reticulum and Golgi apparatus calcium levels.. Cell Calcium.

[38] Kim SK, Park HJ, Hong HS, Baik EJ, Jung MW (2006). ERK1/2 is an endogenous negative regulator of the gamma-secretase activity.. Faseb J.

[39] Baenziger NL, Jong YJ, Yocum SA, Dalemar LR, Wilhelm B (1992). Diversity of B2 bradykinin receptors with nanomolar affinity expressed in passaged IMR90 human lung fibroblasts.. Eur J Cell Biol.

[40] Baenziger NL, Mack P, Jong YJ, Dalemar LR, Perez N (1994). An environmentally regulated receptor for diamine oxidase modulates human endothelial cell/fibroblast histamine degradative uptake.. J Biol Chem.

[41] Wolfe MS (2008). Gamma-secretase: structure, function, and modulation for Alzheimer's disease.. Curr Top Med Chem.

[42] Kornilova A, Das C, Wolfe MS (2003). Differential effects of inhibitors on the gamma-secretase complex: Mechanistic implications.. The Journal of Biological Chemistry.

[43] Saito A, Narasimhan P, Hayashi T, Okuno S, Ferrand-Drake M (2004). Neuroprotective role of a proline-rich Akt substrate in apoptotic neuronal cell death after stroke: relationships with nerve growth factor.. J Neurosci.

[44] Garnier P, Ying W, Swanson RA (2003). Ischemic preconditioning by caspase cleavage of poly(ADP-ribose) polymerase-1.. J Neurosci.

[45] Akao Y, Seki N, Nakagawa Y, Yi H, Matsumoto K (2004). A highly bioactive lidnophenol derivative from bamboo lignin exhibits a potent activity to suppress apoptosis induced by oxidative stress in human neuroblastoma SH-SY5Y cells.. Bioorganic & Medicinal Chemistry.

[46] Lanni C, Uberti D, Racchi M, Govoni S, Memo M (2007). Unfolded p53: a potential biomarker for Alzheimer's disease.. J Alzheimers Dis.

[47] Lanni C, Racchi M, Mazzini G, Ranzenigo A, Polotti R (2008). Conformationally altered p53: a novel Alzheimer's disease marker?. Mol Psychiatry.

[48] Khan TK, Alkon DL (2006). An internally controlled peripheral biomarker for Alzheimer's disease: Erk1 and Erk2 responses to the inflammatory signal bradykinin.. Proc Natl Acad Sci U S A.

[49] Gasparini L, Racchi M, Binetti G, Trabucchi M, Solerte SB (1998). Peripheral markers in testing pathophysiological hypotheses and diagnosing Alzheimer's disease.. Faseb J.

[50] Ray S, Britschgi M, Herbert C, Takeda-Uchimura Y, Boxer A (2007). Classification and prediction of clinical Alzheimer's diagnosis based on plasma signaling proteins.. Nat Med.

[51] Winklhofer KF, Tatzelt J, Haass C (2008). The two faces of protein misfolding: gain- and loss-of-function in neurodegenerative diseases.. Embo J.

[52] Oldenburg O, Qin Q, Krieg T, Yang XM, Philipp S (2004). Bradykinin induces mitochondrial ROS generation via NO, cGMP, PKG, and mitoKATP channel opening and leads to cardioprotection.. Am J Physiol Heart Circ Physiol.

[53] Scheuner D, Eckman C, Jensen M, Song X, Citron M (1996). Secreted amyloid beta-protein similar to that in the senile plaques of Alzheimer's disease is increased in vivo by the presenilin 1 and 2 and APP mutations linked to familial Alzheimer's disease.. Nat Med.

[54] Tan Y, Hong J, Doan T, McConlogue L, Maltese WA (1998). Presenilin-1 mutations associated with familial Alzheimer's disease do not disrupt protein transport from the endoplasmic reticulum to the Golgi apparatus.. Biochim Biophys Acta.

[55] Cheung KH, Shineman D, Muller M, Cardenas C, Mei L (2008). Mechanism of Ca2+ disruption in Alzheimer's disease by presenilin regulation of InsP3 receptor channel gating.. Neuron.

[56] Schroeter EH, Ilagan MX, Brunkan AL, Hecimovic S, Li YM (2003). A presenilin dimer at the core of the gamma-secretase enzyme: insights from parallel analysis of Notch 1 and APP proteolysis.. Proc Natl Acad Sci U S A.

[57] Steiner H (2008). The catalytic core of gamma-secretase: presenilin revisited.. Curr Alzheimer Res.

[58] Yagishita S, Morishima-Kawashima M, Tanimura Y, Ishiura S, Ihara Y (2006). DAPT-induced intracellular accumulations of longer amyloid beta-proteins: further implications for the mechanism of intramembrane cleavage by gamma-secretase.. Biochemistry.

[59] Chen Q, Nakajima A, Choi SH, Xiong X, Tang YP (2008). Loss of presenilin function causes Alzheimer's disease-like neurodegeneration in the mouse.. J Neurosci Res.

[60] Malik B, Currais A, Andres A, Towlson C, Pitsi D (2008). Loss of neuronal cell cycle control as a mechanism of neurodegeneration in the presenilin-1 Alzheimer's disease brain.. Cell Cycle.

[61] Shen J, Kelleher RJ (2007). The presenilin hypothesis of Alzheimer's disease: evidence for a loss-of-function pathogenic mechanism.. Proc Natl Acad Sci U S A.

[62] Nakajima M, Miura M, Aosaki T, Shirasawa T (2001). Deficiency of presenilin-1 increases calcium-dependent vulnerablity of neurons to oxidative stress *in vitro*.. Journal of Neurochemistry.

[63] Brunkan AL, Goate AM (2005). Presenilin function and gamma-secretase activity.. J Neurochem.

[64] Gu F, Zhu M, Shi J, Hu Y, Zhao Z (2008). Enhanced oxidative stress is an early event during development of Alzheimer-like pathologies in presenilin conditional knock-out mice.. Neurosci Lett.

[65] Tu H, Nelson O, Bezprozvanny A, Wang Z, Lee SF (2006). Presenilins form ER Ca2+ leak channels, a function disrupted by familial Alzheimer's disease-linked mutations.. Cell.

[66] Bezprozvanny I, Mattson MP (2008). Neuronal calcium mishandling and the pathogenesis of Alzheimer's disease.. Trends Neurosci.

[67] Smith IF, Hitt B, Green KN, Oddo S, LaFerla FM (2005). Enhanced caffeine-induced Ca2+ release in the 3xTg-AD mouse model of Alzheimer's disease.. J Neurochem.

[68] Boo JH, Sohn JH, Kim JE, Song H, Mook-Jung I (2008). Rac1 changes the substrate specificity of gamma-secretase between amyloid precursor protein and Notch1.. Biochem Biophys Res Commun.

[69] McCarthy JV (2005). Involvement of presenilins in cell-survival signalling pathways.. Biochem Soc Trans.

[70] Cecchi C, Fiorillo C, Sorbi S, Latorraca S, Nacmias B (2002). Oxidative stress and reduced antioxidant defenses in peripheral cells from familial alzheimer's patients.. Free Radical Biology & Medicine.

[71] Shi Q, Gibson GE (2007). Oxidative stress and transcriptional regulation in Alzheimer disease.. Alzheimer Dis Assoc Disord.

[72] Palop JJ, Chin J, Mucke L (2006). A network dysfunction perspective on neurodegenerative diseases.. Nature.

[73] Butterfield D, Boyd-Kimball D (2004). Amyloid beta-peptide (1–42) contributes to the oxidative stress and neurodegeneration found in Alzheimer disease brain.. Brain Pathology.

[74] Biswas SC, Shi Y, Sproul A, Greene LA (2007). Pro-apoptotic Bim induction in response to nerve growth factor deprivation requires simultaneous activation of three different death signaling pathways.. J Biol Chem.

[75] Turner JH, Garnovskaya MN, Raymond JR (2007). Serotonin 5-HT1A receptor stimulates c-Jun N-terminal kinase and induces apoptosis in Chinese hamster ovary fibroblasts.. Biochim Biophys Acta.

[76] Wilhelm M, Xu Z, Kukekov NV, Gire S, Greene LA (2007). Proapoptotic Nix activates the JNK pathway by interacting with POSH and mediates death in a Parkinson disease model.. J Biol Chem.

[77] Beales IL, Ogunwobi O (2006). Glycine-extended gastrin inhibits apoptosis in colon cancer cells via separate activation of Akt and JNK pathways.. Mol Cell Endocrinol.

[78] Johnstone ED, Mackova M, Das S, Payne SG, Lowen B (2005). Multiple anti-apoptotic pathways stimulated by EGF in cytotrophoblasts.. Placenta.

[79] Lin A (2003). Activation of the JNK signaling pathway: breaking the brake on apoptosis.. Bioessays.

[80] Zhu X, Castellani RJ, Takeda A, Nunomura A, Atwood CS (2001). Differential activation of neuronal ERK, JNK/SAPK and p38 in Alzheimer disease: the ‘two hit’ hypothesis.. Mechanisms of Ageing and Development.

[81] Zhu X, Rottkamp CA, Aliev G, Perry G, Boux H (2001). Activation and redistribution of c-Jun N-terminal kinase/stress activated protein kinase in degenerating neurons in Alzheimer's disease.. Journal of Neurochemistry.

[82] Li Y, Rowland C, Catanese J, Morris J, Lovestone S (2008). SORL1 variants and risk of late-onset Alzheimer's disease.. Neurobiol Dis.

[83] Mukherjee O, Kauwe JS, Mayo K, Morris JC, Goate AM (2007). Haplotype-based association analysis of the MAPT locus in late onset Alzheimer's disease.. BMC Genet.

[84] Kauwe JS, Cruchaga C, Mayo K, Fenoglio C, Bertelsen S (2008). Variation in MAPT is associated with cerebrospinal fluid tau levels in the presence of amyloid-beta deposition.. Proc Natl Acad Sci U S A.

[85] Kauwe JS, Jacquart S, Chakraverty S, Wang J, Mayo K (2007). Extreme cerebrospinal fluid amyloid beta levels identify family with late-onset Alzheimer's disease presenilin 1 mutation.. Ann Neurol.

[86] Ringman JM, Younkin SG, Pratico D, Seltzer W, Cole GM (2008). Biochemical markers in persons with preclinical familial Alzheimer disease.. Neurology.

[87] Quinn J, Sabbagh MN (2008). Detecting Alzheimer disease before it happens: The key to prevention?. Neurology.

[88] Bateman RJ, Munsell LY, Morris JC, Swarm R, Yarasheski KE (2006). Human amyloid-beta synthesis and clearance rates as measured in cerebrospinal fluid in vivo.. Nat Med.

[89] Costanzi E, Martino S, Persichetti E, Tiribuzi R, Massini C (2007). Effects of Vitamin C on Fibroblasts from Sporadic Alzheimer's Disease Patients.. Neurochem Res.

[90] Bellucci C, Lilli C, Baroni T, Parnetti L, Sorbi S (2007). Differences in extracellular matrix production and basic fibroblast growth factor response in skin fibroblasts from sporadic and familial Alzheimer's disease.. Mol Med.

[91] Combarros O, Cortina-Borja M, Smith AD, Lehmann DJ (2008). Epistasis in sporadic Alzheimer's disease.. Neurobiol Aging.

[92] Fagan AM, Csernansky CA, Morris JC, Holtzman DM (2005). The search for antecedent biomarkers of Alzheimer's disease.. J Alzheimers Dis.

[93] Fagan AM, Roe CM, Xiong C, Mintun MA, Morris JC (2007). Cerebrospinal fluid tau/beta-amyloid(42) ratio as a prediction of cognitive decline in nondemented older adults.. Arch Neurol.

[94] Borroni B, Premi E, Di Luca M, Padovani A (2007). Combined biomarkers for early Alzheimer disease diagnosis.. Curr Med Chem.

[95] Lee JM, Blennow K, Andreasen N, Laterza O, Modur V (2008). The Brain Injury Biomarker VLP-1 Is Increased in the Cerebrospinal Fluid of Alzheimer's Disease Patients.. Clin Chem.

[96] Park IH, Zhao R, West JA, Yabuuchi A, Huo H (2008). Reprogramming of human somatic cells to pluripotency with defined factors.. Nature.

[97] Dimos JT, Rodolfa KT, Niakan KK, Weisenthal LM, Mitsumoto H (2008). Induced Pluripotent Stem Cells Generated from Patients with ALS Can Be Differentiated into Motor Neurons.. Science.

